# Tiled vector data model for the geographical features of symbolized maps

**DOI:** 10.1371/journal.pone.0176387

**Published:** 2017-05-05

**Authors:** Lin Li, Wei Hu, Haihong Zhu, You Li, Hang Zhang

**Affiliations:** 1 School of Resource and Environmental Sciences, Wuhan University, Wuhan, China; 2 Geo Spatial Information Science Collaborative Innovation Center of Wuhan University, Wuhan, China; 3 The Key Laboratory for Geographical Information Systems, Ministry of Education, Wuhan, China; Agricultural University of Athens, GREECE

## Abstract

Electronic maps (E-maps) provide people with convenience in real-world space. Although web map services can display maps on screens, a more important function is their ability to access geographical features. An E-map that is based on raster tiles is inferior to vector tiles in terms of interactive ability because vector maps provide a convenient and effective method to access and manipulate web map features. However, the critical issue regarding rendering tiled vector maps is that geographical features that are rendered in the form of map symbols via vector tiles may cause visual discontinuities, such as graphic conflicts and losses of data around the borders of tiles, which likely represent the main obstacles to exploring vector map tiles on the web. This paper proposes a tiled vector data model for geographical features in symbolized maps that considers the relationships among geographical features, symbol representations and map renderings. This model presents a method to tailor geographical features in terms of map symbols and ‘addition’ (join) operations on the following two levels: geographical features and map features. Thus, these maps can resolve the visual discontinuity problem based on the proposed model without weakening the interactivity of vector maps. The proposed model is validated by two map data sets, and the results demonstrate that the rendered (symbolized) web maps present smooth visual continuity.

## Introduction

Web electronic maps (E-maps) have been extensively exploited and widely applied with the rapid development of technologies such as wireless communication, computer technology and geographic space information technology [1]. Many successful applications of E-maps have been implemented in daily life [2]. E-maps can be used as information inquiry services by providing users with specific information, such as POIs (points of interest) and tourist information [3,4]. E-maps present bus routes and real-time traffic information and provide data that support vehicle navigation [5,6]. E-maps are also useful for teaching geography [7] and urban planning [8]. The emergence of social media (such as Twitter, Facebook, and the Sina microblog) has inspired a considerable amount of research that has used check-in data and additional data regarding the social ties between users, which are collected from various sources [9]. Street views [10], indoor maps [11] and WEBGIS/VR [12–15] further extend the applications of E-maps.

The traditional applications of E-maps are implemented based on web map services (WMS) [16]. However, the spatial parameters in WMS requests are not constrained, which forces images to be generated on the fly each time a request is received, and the processes involved in the image generation by the WMS server cause delays typically on the order of several seconds [17]. Furthermore, WMS is not scalable to large numbers of users due to a lack of ability to cache requests and this is implicit in the way the standards are written [18]. Therefore, Google Maps, Yahoo Maps, Virtual Earth and other popular E-maps use a “pyramid technique” that is based on tiles in the form of raster data to improve transmission performance [19]. A new map service standard called Web Map Tile Service (WMTS) has been developed based on WMS and the “pyramid technique”, which have been adopted by most large-scale web mapping systems [17,18,20]. Under the WMTS framework, the map is divided into images called “tiles” that are transmitted to the client side according to the requested region. The tiles are re-combined by using their respective coordinates on the client side [21–24].

Another critical issue for E-maps is interactivity, which is also a hallmark of E-maps [25]. E-maps with broader interactive functions can enable users to use the map data for spatial analyses and temporal-spatial phenomena investigations [26–30]. Interactive E-maps can help strengthen spatial cognition, enhance expressive abilities and improve map availability [26,31–33].

Compared with raster data maps, which are limited in their interactivity, maps that use vector data provide excellent and unique advantages in accessing geographical features, although the uneven data amounts of vector maps may cause transmission efficiency problems. The Open Geospatial Consortium provides a Web Feature Service Interface Standard (WFS), which enables requests for geographical features across the web. To overcome the lower vector data transmission rates, many studies have proposed progressive transmission solutions [34–39] and data compression to increase transmission efficiency [40–43]. Meanwhile, the emergence of GeoJSON has provided a better web data encoding format to improve transmission efficiency [44]. Compared to XML-based web data encoding formats (GML/KML), GeoJSON can be more easily and quickly parsed by a computer, and can describe complex data structures [43]. GeoJSON also provides a light-weight data encoding format that can be easily transferred. These efforts have indeed improved the performance of vector map transmissions over the Internet. However, transmitting all vector data online is still time consuming and likely intolerable for interactive operations, and the pyramid technique remains an effective organizing method for vector maps, namely vector tile map.

When a vector map is presented on the client side, individual map tiles are sequentially transmitted through the Internet and then reassembled into a map. To decrease the amount of transmitted data, a vector map can be divided into two data sets: geographical features and cartographic representation. Only geographical features are transmitted, and cartographical representation is completed locally on the client side. Cartographical representation includes the rules for portraying geographical features and map symbols [45]. For example, an ArcGIS vector map is represented by geographic features and *.style files [46,47]. Map legibility largely depends on the rendering of map features with different symbols [48]. However, this organizational structure of vector maps is challenged when geographical features are symbolized in individual ‘vector tiles’ and joined with neighboring features. Such a map rendering method produces visual discontinuities and conflicts at the tile borders, which may break the spatial distribution characteristics of features [49] and reduce the readability and applicability of the map [48,50]. Therefore, a critical issue for tiled vector E-maps is to join neighboring features with their symbol representation to maintain visual continuity and avoid visual conflicts.

To eliminate visual breakage, this study proposes a tiled vector data model for the geographical features that define the additivity of map features and geographical features, partition vector geographical features, and implement map symbolizations to graphically match joined symbolized partitioned features without causing graphic conflicts and losses.

This paper is organized as follows: Section 2 describes the symbol representation method. Section 3 introduces the tiled vector data model, verifies the feasibility of the data model, and then proposes different data organization methods for geographical features based on the tiled vector data model. Section 4 reports an experiment that uses the proposed model and discusses the results of the experiment. Finally, Section 5 presents the conclusions and future work.

## Symbol representation

**Fig 1:**
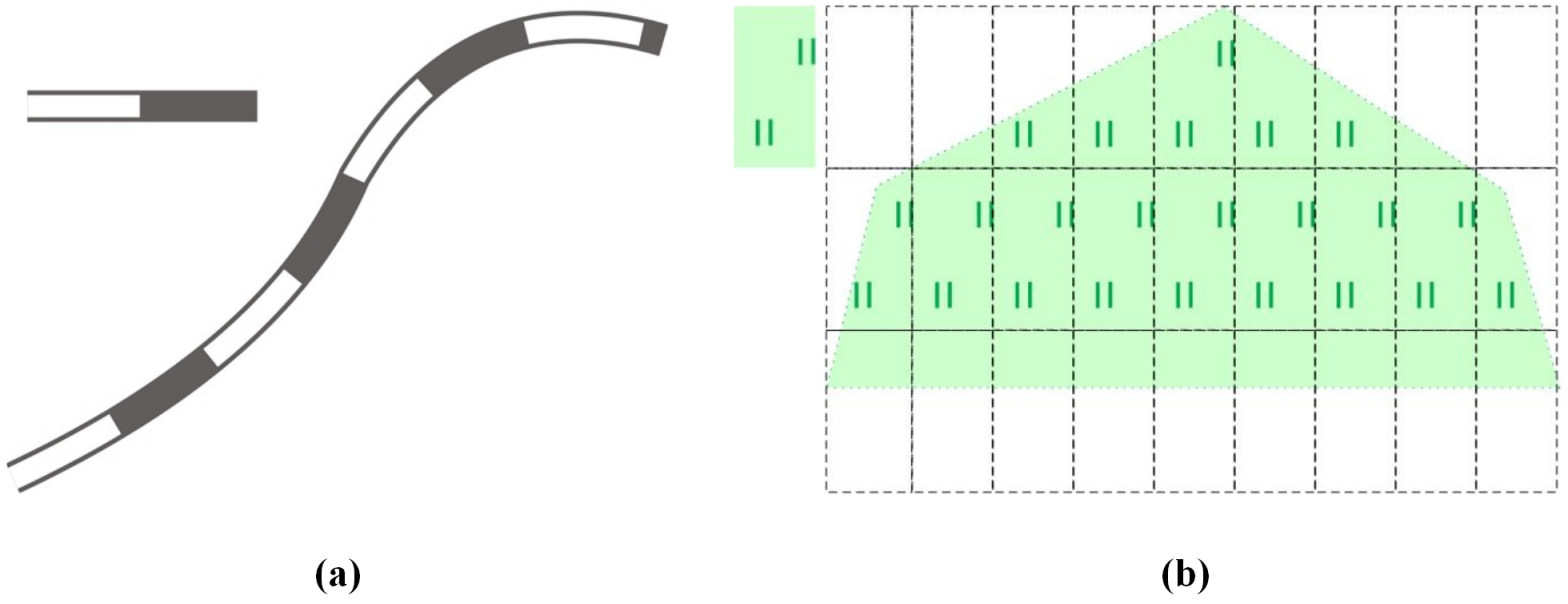
(a) Railway symbol and its map feature, and (b) grassland symbol and its map feature.

Point symbols, linear symbols and area symbols are the three basic symbol representation methods for vector data in cartography and geographic information system [48]. The symbol representation for point features renders the specified point symbol at a location point. The symbol representation for area features can be summarized as the rendered filling of different graphic cells based on the scan line algorithm or its improved algorithms. Compared to the symbol representation for point symbols and area symbols, the symbol representation for linear features is much more complicated. A linear feature is generally symbolized by repeatedly drawing the corresponding symbol along its path. Fig 1(a) shows the symbol of a railway and the map feature of a railway geographical feature (representing the linear feature), and Fig 1(b) shows the symbol of grassland and the map feature of a grassland geographical feature (representing the area feature).

Any symbol *q* in the symbol library *Q* is an ordered set that consists of geometric graphics (such as polylines, polygons and ellipses) with properties that include color and line width. The circumscribing polygon of the symbol can be expressed as a rectangle; the width of the rectangle is referred to as the width of the symbol, and the length of the rectangle is referred to as the length of the symbol. *λ* is a symbol parameter: when the feature is a linear feature, the symbol parameter *λ* denotes the length of the linear symbol *l*, and when the feature is an area feature, *λ* denotes the width of the symbol *w* and the length *l* of the symbol [51]. The symbol parameter *λ* can thus be expressed as follows:

$$\lambda =\{\begin{array}{r} \begin{array}{r} l,\;\text{the symbol is a linear symbol}\\ \left(w,l\right),\;\text{the symbol is an area symbol} \end{array} \end{array}$$ (1)

## Tiled vector data model for geographical features

### Mapping geographical feature space on map feature space

Geographical feature space is the abstract expression of spatial entities and geographical phenomena in the real world [52]. Geographical feature space can be converted to map feature space by symbol representation. This conversion process can be expressed as follows [51]:

M=S(G,Q) (2)

where *M* represents the map feature space, *G* represents the geographical feature space, *Q* represents the symbol library (set) that corresponds to the geographical feature space *G*, and *S* represents the common function for symbolizing the geographical feature space. The conversion process for an individual map feature in the map feature space can be expressed as follows:

m=S(g,q)(m∈M,g∈G,q∈Q) (3)

In eq (3), the connection between geographic features and map features is fixed for a given scale, this is because the symbol is fixed. However, a feature may have different symbols at different scales.

### Graphic conflicts in symbolization and operators for tiled vector data model

**Fig 2:**
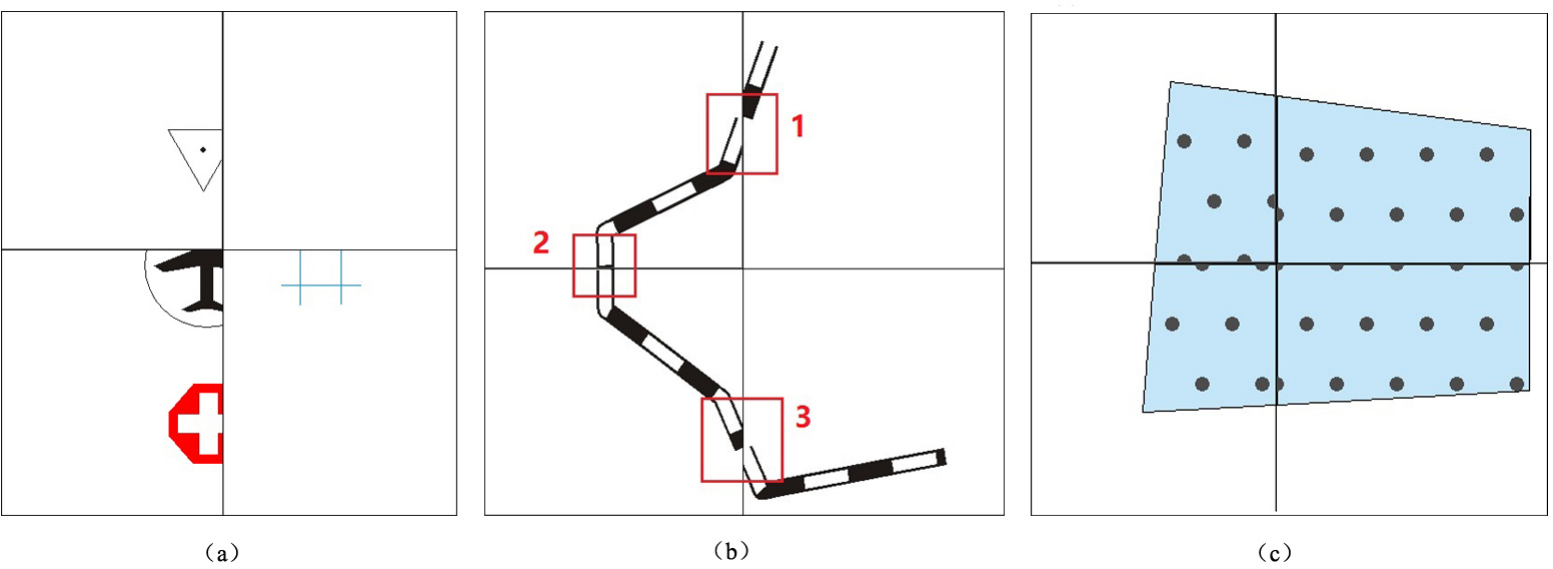
Graphic conflicts and losses.

Graphic conflicts and losses may occur along the borders of tiles when symbolizing geographical features on each tile and connecting tiles according to their respective coordinates. Examples of the three basic types of problems that arise are shown in Fig 2. As shown in Fig 2(a), when the points at the borders of the tiles are symbolized, the components of the map features inside the corresponding tile are easily rendered. Nonetheless, we cannot omit the components of map features that are outside these tiles because such deletions would create additional issues, such as a loss of certain components and the non-conformity of map features along the tile borders. Fig 2(b) shows an example of symbolizing a linear feature along the borders of these tiles, and the problems are marked. The loss of map features from Mark 1 and Mark 3 is obvious, and graphic conflicts between two adjacent tiles are shown at Mark 2. Fig 2(c) shows graphic conflicts in area map features that are caused by filling graphics cells at neighboring tiles. The reason for the graphic conflicts in Fig 2 is that geographical features are clipped into different vector tiles: when symbolizing the clipped features on each tile and connecting tiles according to their respective coordinates, the symbolized features (map features) cannot match well at the tile borders. Thus, obvious graphic conflicts occur between map features in neighboring tiles. The key to resolving these problems is to design or construct an operator for map features with ‘additivity’, which means that two separate symbolizations can yield the same graphic presentation as a single symbolization.

Several basic concepts and signs are introduced here to illustrate the above problems and clarify the principle of the proposed model. The “addition” sign ⊕ denotes joining two features, ⊕_*M*_ denotes joining two map features and ⊕_*G*_ denotes joining two geographical features.

**Definition 1.** The premise of the “addition” operator for geographical features: The operation of *g*_1_⊕_*G*_*g*_2_ is tenable if EITHER (1) both *g*_1_ and *g*_2_ are linear features, *g*_1_ and *g*_2_ are connected at their ends, or *g*_1_ = *g*_2_; OR (2) both *g*_1_ and *g*_2_ are area features and *g*_1_ intersects, touches or contains *g*_2_.

**Fig 3:**
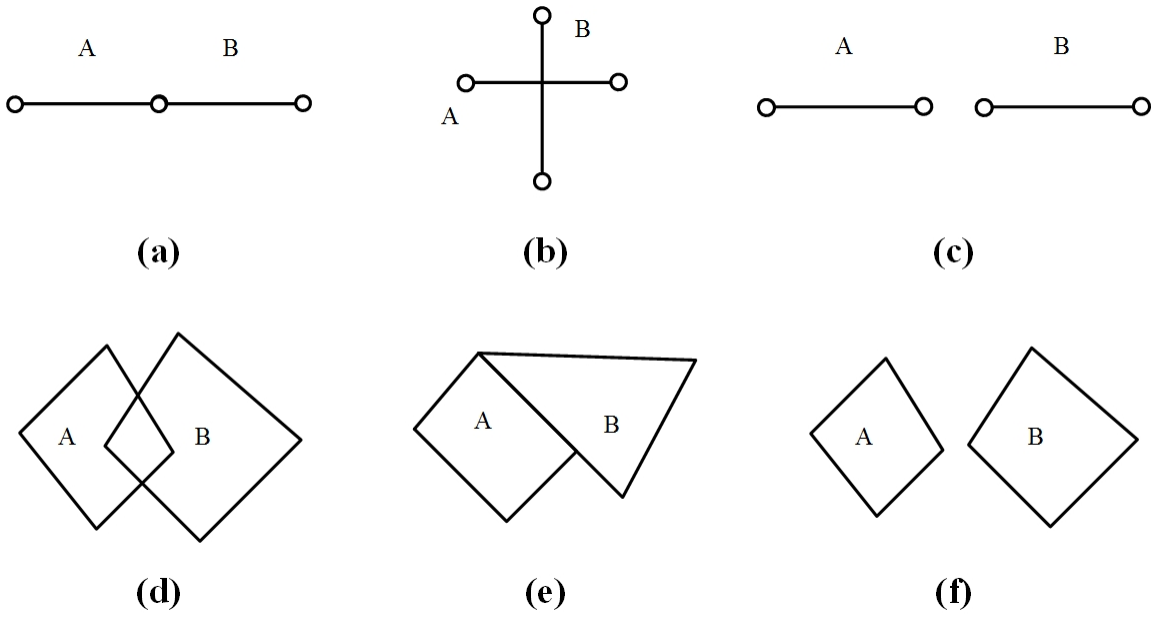
Examples of the premise of the “addition” operator for geographical features.

Examples for Definition 1 are shown in Fig 3, where (a), (d) and (e) satisfy the premise of the “addition” operator for geographical features and (b), (c) and (f) do not satisfy the premise of the “addition” operator.

**Definition 2.** The “accumulation” operator for geographical features: An “accumulation” operator for geographical features is defined as **∧**. $\wedge_{i=1}^{n}g_{i}$ can be expressed as $\wedge_{i=1}^{n}g_{i}\;=\;g_{1}{⊕}_{G}g_{2}{⊕}_{G}\;\ldots \;{⊕}_{G}g_{n}$(*g*_*i*_ ∈ G, n ≥ 1, *g*_1_.*g*_2_ … *g*_*n*_ satisfy Definition 1).

**Fig 4:**
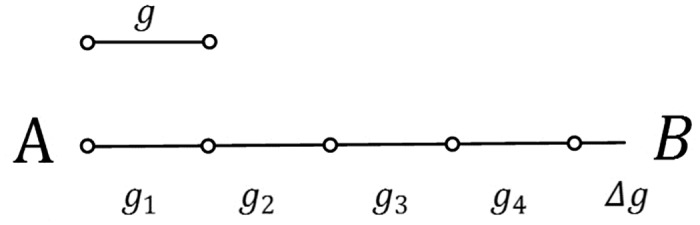
Examples of the “accumulation” operator for geographical features.

The difference between the “addition” sign for geographical features and the “accumulation” operator in this study is similar to the difference between the plus sign “+” and the sum operator “∑” in mathematics. To avoid cognitive conflicts with common mathematical operators, we use “⊕_*G*_” and “∧” to replace “+” and “∑”, respectively. The “accumulation” operator for geographical features is used in this article to simplify this formula. As shown in Fig 4, *g* is a symbol, and the linear feature A-B is divided into 5 components (*g*_1_, *g*_2_, *g*_3_, *g*_4_ and Δ*g*) by this symbol. Thus, the linear feature can be written as *g*_1_⊕_*G*_*g*_2_⊕_*G*_*g*_3_⊕_*G*_*g*_4_⊕_*G*_Δ*g*. However, expressing all the geographical features in this manuscript similarly to this formula is tedious. Therefore, we use the “accumulation” operator to simplify this formula to $\wedge_{i=1}^{4}g_{i}\;{⊕}_{G}\Delta g$.

**Definition 3.** The premise of the “addition” operator for map features: The geographical features *g*_1_ and *g*_2_ have the same symbol *q* and a corresponding symbol parameter *λ*. By using these symbols, we can obtain the map features *m*_1_ = *S*(*g*_1_, *q*) and *m*_2_ = *S*(*g*_2_, *q*). The formula *m*_1_⊕_M_*m*_2_ = *m*_3_ holds true when (1) feature *g*_1_ can be divided into *n* components ($g_{1}^{\lambda },g_{2}^{\lambda }\ldots g_{n}^{\lambda }$, where *n* is an integer) by the symbol parameter *λ* and (2) *g*_1_ and *g*_2_ satisfy Definition 1. In this formula, *m*_3_ can be expressed as *m*_3_ = *S*(*g*_1_⊕_*G*_*g*_2_, *q*).

**Fig 5:**
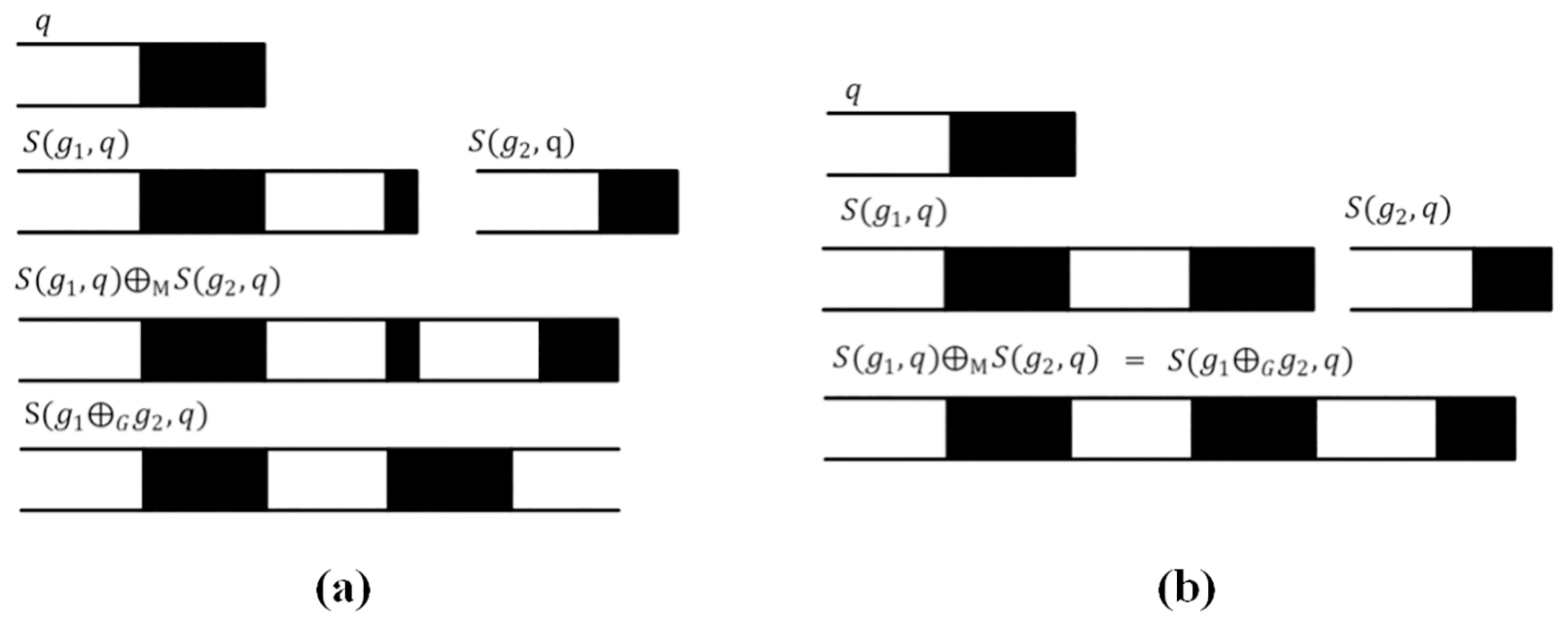
Examples of the premise of the “addition” operator for map features.

If the features satisfy Definition 3, the map features can match well. Some examples are shown in Fig 5 to explain Definition 3. In Fig 5, both *g*_1_ and *g*_2_ are the railway feature, and *q* is the railway symbol. *S*(*g*_1_, *q*) and *S*(*g*_2_, *q*) do not satisfy the premise of the “addition” operator for map features in Fig 5(a) because feature *g*_1_ cannot be divided into n components (n is an integer) by the length of the symbol *q*. *S*(*g*_1_, *q*)⊕_M_ *S*(*g*_2_, *q*) is obviously not equal to *S*(*g*_1_⊕_*G*_*g*_2_, *q*). In Fig 5(b), feature *g*_1_ can be divided into n components (n is an integer) by the length of the symbol *q*, and *S*(*g*_1_, *q*)⊕_M_ *S*(*g*_2_, *q*) is equal to *S*(*g*_1_⊕_*G*_*g*_2_, *q*).

**Definition 4.** The invariance of addition by itself: For any feature *φ*(*φ* ∈ M ‖ *φ* ∈ *G*), *φ*⊕*φ = φ* is tenable.

**Fig 6:**
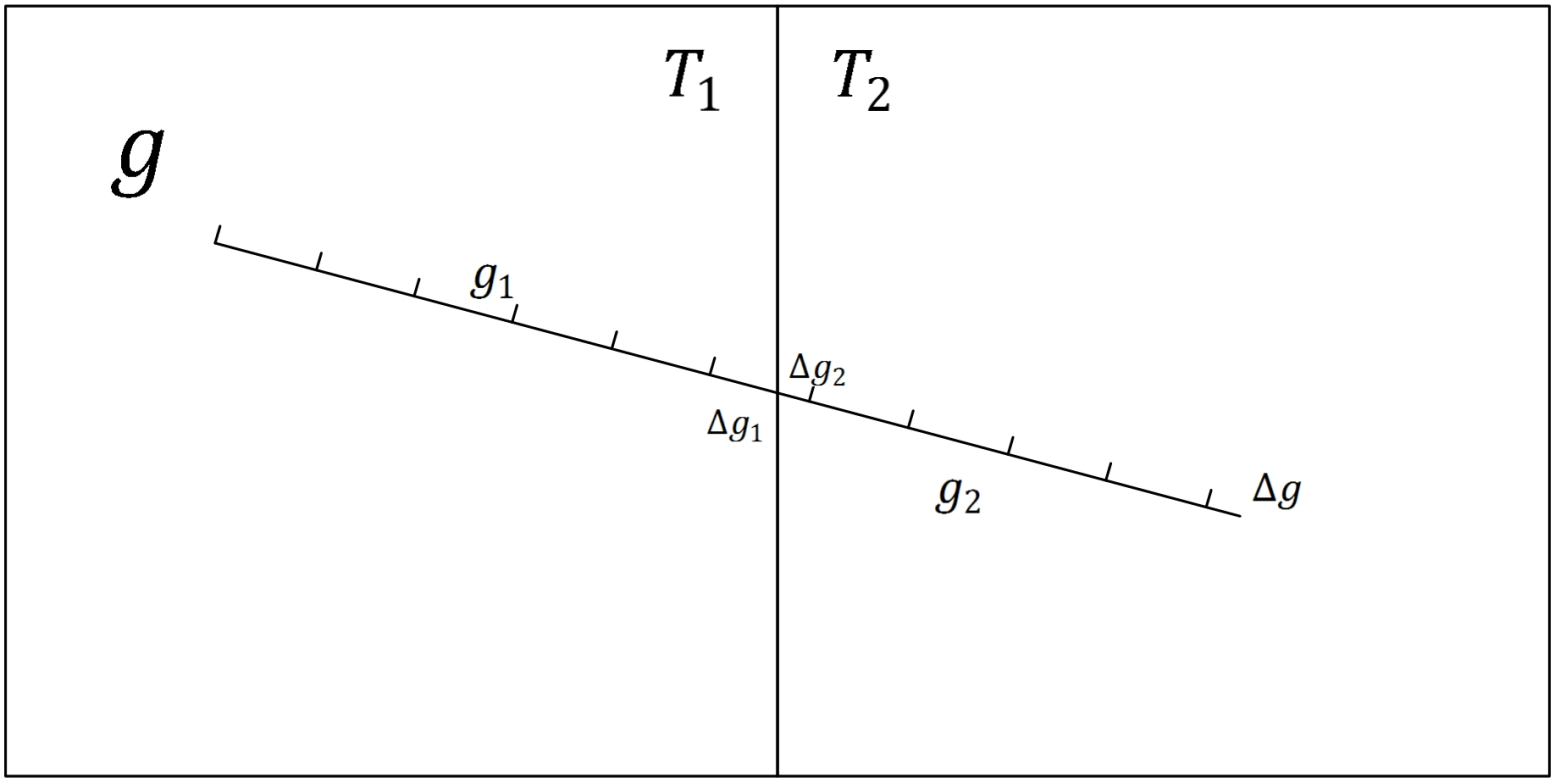
Example of linear features.

The railway symbol is chosen as an example to illustrate why the traditional vector data model is already inapplicable to vector map tiles based on Definitions 1–4 but applicable for other linear symbols. For example, Fig 6 shows two tiles called *T*_1_ and *T*_2_; a railway feature *g* crosses the tiles and is clipped by the tiles into *g*_1_ and *g*_2_. Thus, we can obtain the following formula:

$$g=g_{1}{⊕}_{G}g_{2}$$ (4)

The map feature that corresponds to *g* can be expressed as follows:

m=S(g,q) (5)

where *q* is the railway symbol and *m* denotes the map feature of *g*. Similarly, the map features of *g*_1_ and *g*_2_ can be represented by the following formulas:

$$m_{1}=S\left(g_{1},q\right)$$ (6)

$$m_{2}=S\left(g_{2},q\right)$$ (7)

If *m*_1_⊕_M_*m*_2_ is equal to *m*, then the map features match well at the borders of the tiles. As shown in Fig 6, we introduce *λ* to represent the length of the symbol *q*, and then feature *g* can be divided into *n* components ($g_{1}^{\lambda },g_{2}^{\lambda }\ldots g_{n}^{\lambda }$, where *n* is an integer) by the corresponding symbol parameter *λ*. As shown in Fig 6, we assume that feature *g* intersects tiles at the component *k* of the feature and that the tiles divide $g_{k}^{\lambda }$ into Δ*g*_1_ and Δ*g*_2_. Δ*g*_1_ and Δ*g*_2_ are located in *T*_1_ and *T*_2_, respectively. Feature g can then be rewritten as follows:

$$g=\wedge_{i=1}^{n}g_{i}^{\lambda }{⊕}_{G}\Delta g$$ (8)

The expression of feature *g*_1_ then becomes:

$$g_{1}=\wedge_{i=1}^{k-1}g_{i}^{\lambda }{⊕}_{G}\Delta g_{1}$$ (9)

The expression of feature *g*_2_ then becomes:

$$g_{2}=\Delta g_{2}{⊕}_{G}\wedge_{i=k+1}^{n}g_{i}^{\lambda }{⊕}_{G}\Delta g$$ (10)

**Fig 7:**
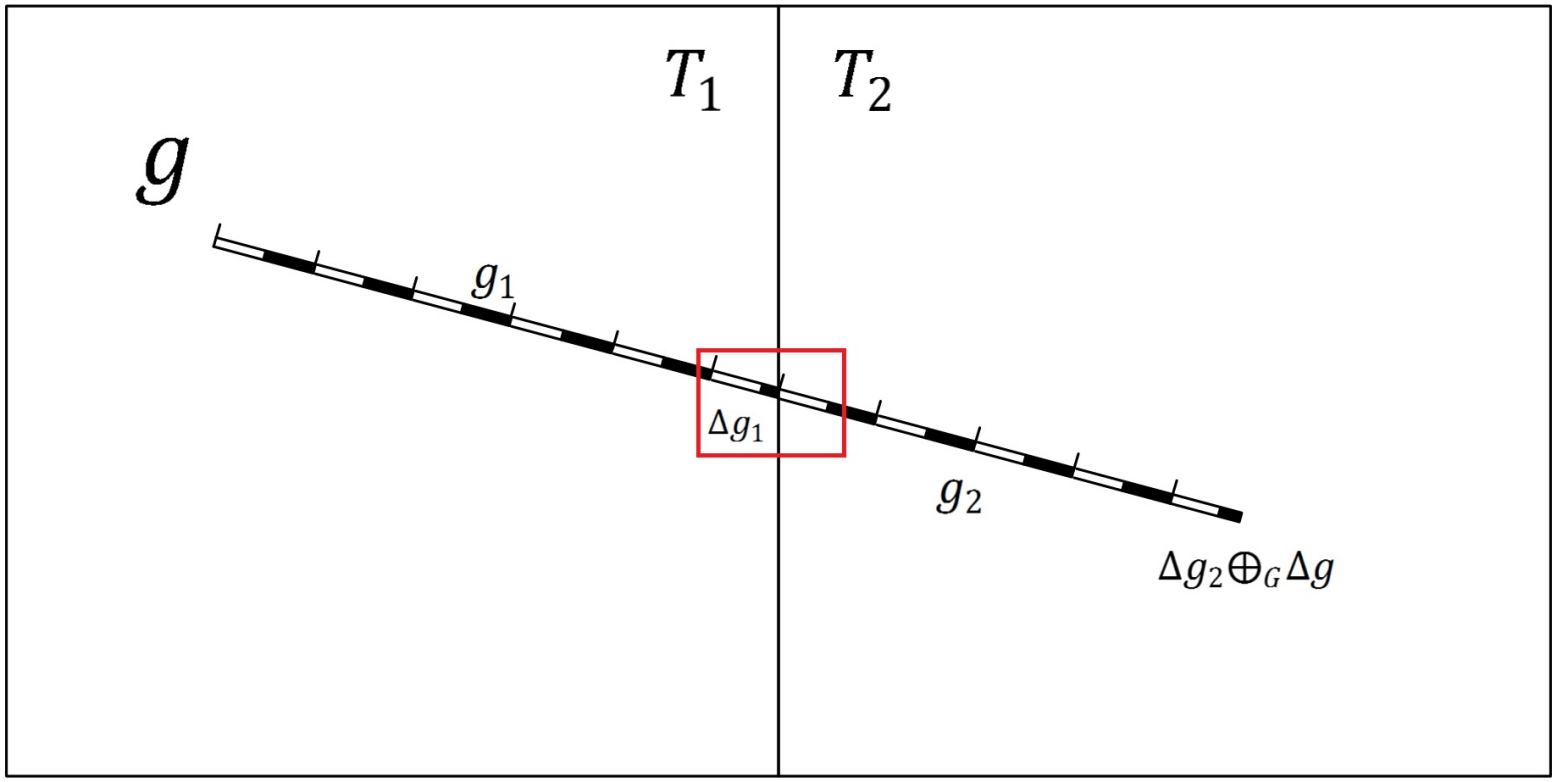
Example of linear features based on the traditional data model.

In Formula (9), *Δg*_1_ is not equal to *g*^*λ*^, as shown in Fig 6, so *g*_1_ cannot be divided into n components (n is an integer) by the length of the symbol *q*. Therefore, *m*_1_⊕_M_*m*_2_ does not satisfy Definition 3. As a result, *m*_1_⊕_M_*m*_2_ is not equal to *m* and the map features of feature *g*_1_ and feature *g*_2_ do not match well at the borders of the tiles, an illustrative diagram is shown as Fig 7.

Both linear features (railway) and other types of features encounter this problem. Therefore, the key to implementing the vector map tile technique is proposing a tiled vector data model for geographical features so that tiles can comprise an entire map with accurate and complete cartographic representation. Using the proposed data model can make the cartographic representation results render more favorably for features along the borders of tiles. Meanwhile, the rationality of the data model is also verified in this section.

### Tiled vector data models for point, linear and area features

We designed a novel tiled vector data model for all types of features to match map features well along the borders of tiles.

#### Tiled vector data model for linear features

A tiled vector data model for linear features is proposed based on Definition 3 as follows:

$$g=\{\begin{array}{lll} \wedge_{i=1}^{n}g_{i}^{\lambda }{⊕}_{G}\Delta g{⊕}_{G}\sigma , & \text{Case 1:} & \text{The end point of the linear feature is the intersection point.}\\ \sigma {⊕}_{G}\Delta g_{1}{⊕}_{G}\wedge_{i=1}^{n}g_{i}^{\lambda }{⊕}_{G}\Delta g, & \text{Case 2:} & \text{The start point of the linear feature is the intersection point.}\\ \sigma_{1}{⊕}_{G}\Delta g_{1}{⊕}_{G}\wedge_{i=1}^{n}g_{i}^{\lambda }{⊕}_{G}\Delta g_{2}{⊕}_{G}\sigma_{2}, & \text{Case 3:} & \text{The start point and end point of the linear feature are the intersection points.} \end{array}$$ (11)

**Fig 8:**
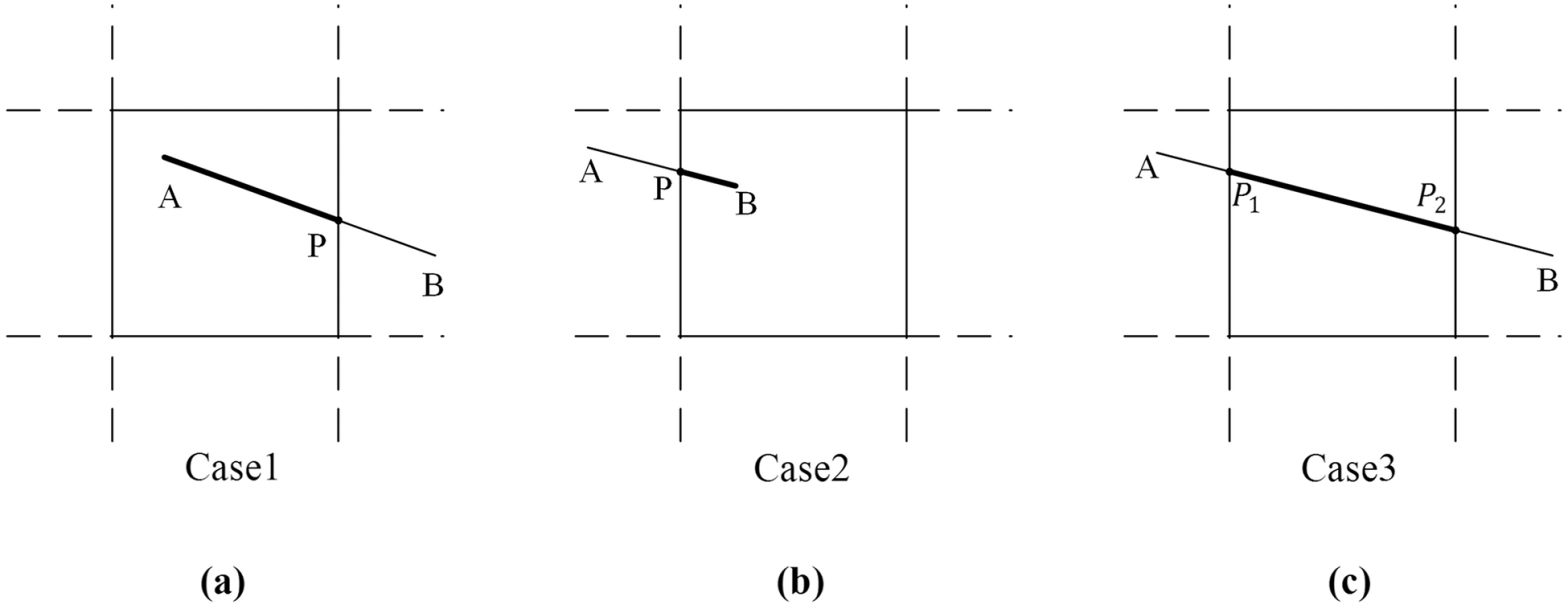
Examples of linear features for the tiled vector data model.

In Formula (11), *σ* is an expansion feature and *Δg*⊕_*G*_*σ* = *g*^***λ***^. Fig 8 shows examples for Formula (11). In Fig 8(a), the linear feature A-B intersects the tile at point *P*. The end point for the linear feature A-P is the intersection point, therefore, the data model for feature A-P is case 1 in Formula (11). In Fig 8(b), the data model for feature P-B is case 2 in Formula (11). In Fig 8(c), the data model for feature P_1_-P_2_ is case 3 in Formula (11).

**Fig 9:**
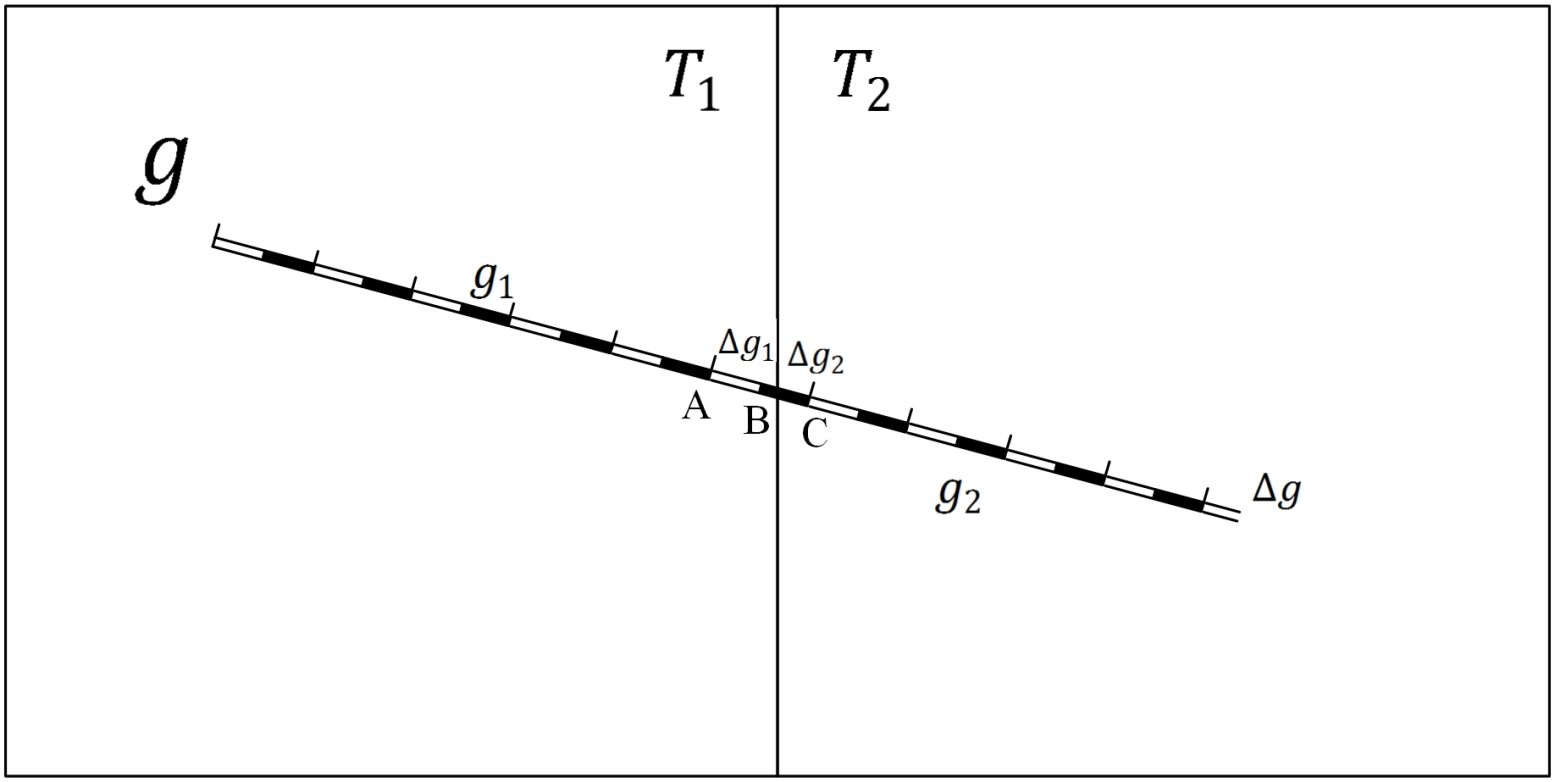
Example of linear features based on the tiled vector data model.

An illustrative diagram that is based on the proposed tiled vector data model for linear features in Fig 6 is shown in Fig 9. Based on Formula (11), the feature *g*_1_ in Fig 6 satisfies case 1 and thus can be changed as follows:

$$g_{1}=\wedge_{i=1}^{k-1}g_{i}^{\lambda }{⊕}_{G}\Delta g_{1}{⊕}_{G}\Delta g_{2}=\wedge_{i=1}^{k-1}g_{i}^{\lambda }{⊕}_{G}g_{k}^{\lambda }$$ (12)

The feature *g*_2_ in Fig 6 satisfies case 2 and can be rewritten as follows:

$$g_{2}=\Delta g_{1}{⊕}_{G}\Delta g_{2}{⊕}_{G}\wedge_{i=k+1}^{n}g_{i}^{\lambda }{⊕}_{G}\Delta g=g_{k}^{\lambda }{⊕}_{G}\wedge_{i=k+1}^{n}g_{i}^{\lambda }{⊕}_{G}\Delta g$$ (13)

According to Formula (12) and Formula (13), the expansion features of *g*_1_ and *g*_2_ are Δ*g*_2_ and Δ*g*_1_, respectively, and *m*_1_⊕_M_*m*_2_ can be rewritten as follows:

$$\begin{array}{c} m_{1}{⊕}_{\text{M}}m_{2}=\left(S\left(\wedge_{i=1}^{k-1}g_{i}^{\lambda },q\right){⊕}_{\text{M}}S\left(g_{k}^{\lambda },q\right)\right){⊕}_{\text{M}}(S\left(g_{k}^{\lambda },q\right){⊕}_{\text{M}}\\ S\left(\wedge_{i=k+1}^{n}g_{i}^{\lambda },q\right){⊕}_{\text{M}}S\left(\Delta g,q\right)) \end{array}$$ (14)

Formula (14) can be simplified as follows based on Definition 3 and Definition 4:

$$\begin{array}{ll} m_{1}{⊕}_{\text{M}}m_{2} & =S\left(\wedge_{i=1}^{k-1}g_{i}^{\lambda },q\right){⊕}_{\text{M}}S(g_{k}^{\lambda },q){⊕}_{\text{M}}S\left(\wedge_{i=k+1}^{n}g_{i}^{\lambda },q\right){⊕}_{\text{M}}S\left(\Delta g,q\right)\\ & =S\left(\wedge_{i=1}^{n}g_{i}^{\lambda },q\right){⊕}_{\text{M}}S\left(\Delta g,q\right) \end{array}$$ (15)

In Formula (15), *m*_1_⊕_M_*m*_2_ is equal to *m*, which means that using the data model for linear features in Formula (11) can solve the problems of graphic conflicts and losses at the borders of tiles. This relationship also proves that expanding a feature that is clipped by a tile to a complete symbol can resolve these problems.

#### Tiled vector data model for point features

When using the method of linear features for reference and by considering the simplicity of point features, the data model for point features is as follows:

$$g=\{\begin{array}{lll} g, & \text{Case 1:} & \text{The tile contains point. }\\ \sigma , & \text{Case 2:} & \text{The tile does not contains point , the map feature intersects the tile.}\\ \emptyset , & \text{Case 3:} & \text{The map feature does not intersect the tile}. \end{array}$$ (16)

In the data model, *σ* is an expansion feature and *σ* = *g*. According to Formula (16), case 1 means that if the tile contains a point feature, this point feature is added to the feature set of the tile. In case 2, when the tile does not contain a point feature but the map feature of a point intersects the tile, we also add the point feature to the feature set of the tile. In case 3, when the map feature of a point does not intersect the tile, then the model skips the point feature.

#### Tiled vector data model for area features

Research on the rendering of area symbols can be summarized as the filling of different graphic cells [48]. Graphic cells have two patterns: color filling and symbol filling. When the filling pattern is color filling, the map features of an area feature can match well along the borders of tiles. However, when the filling pattern is symbol filling, as shown in Fig 2(c), then obvious graphic conflicts from area map features occur within neighboring tiles. Generally, a line is considered a one-dimensional object and an area is considered a two-dimensional object. Area features are more complicated than linear features; specifically, the tiled vector data model for linear features cannot adequately handle area features. We now introduce several basic concepts and signs prior to introducing the tiled vector data model for area features.

**Definition 5.** The symbolization starting point of area features: The upper-left corner of the circumscribing rectangle of an area feature is called the symbolization starting point of the area feature.

**Fig 10:**
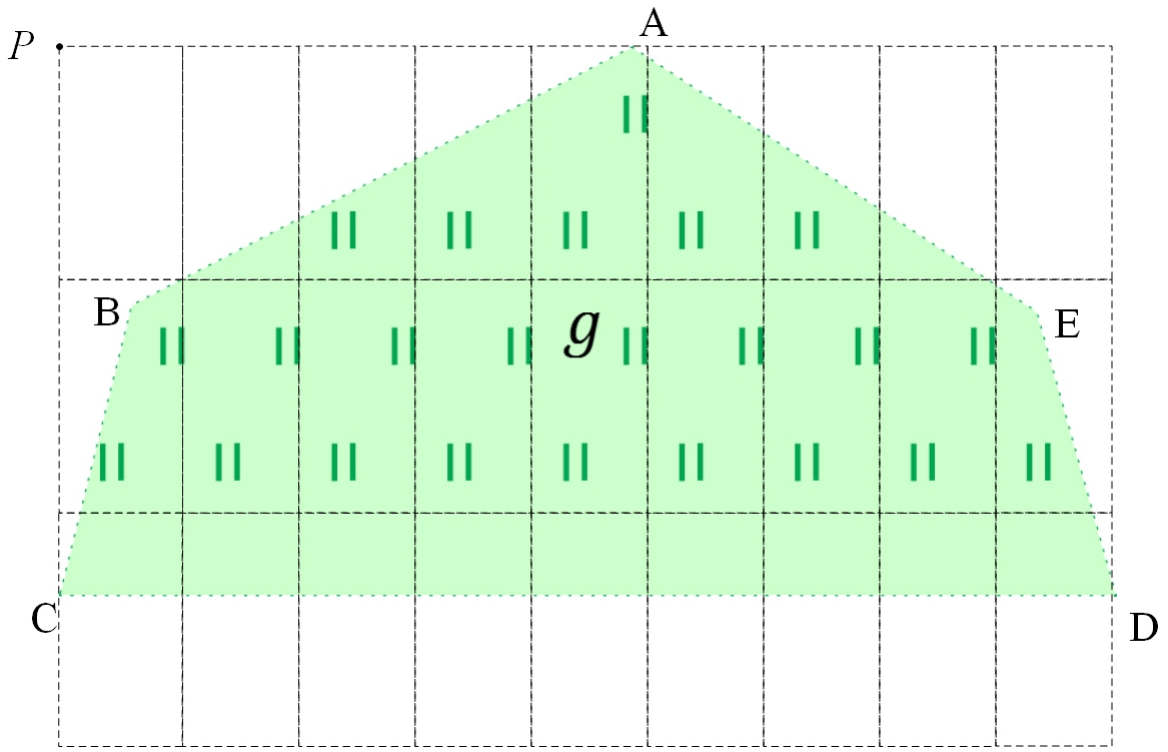
Examples of symbolization starting points for area features.

As shown in Fig 10, the upper-left corner of the circumscribing rectangle of area feature *g* (polygon A-B-C-D-E) is vertex P, so vertex P is called the symbolization starting point of feature *g*.

**Definition 6.** The premise of the “addition” operator for area map features: For area features *g*_1_ and *g*_2_, the symbolization starting points of *g*_1_ and *g*_2_ are located at *P*_1_(*x*_1_, *y*_1_) and *P*_2_(*x*_2_, *y*_2_), respectively. These features have the same symbol *q*, and the corresponding symbol parameter *λ* contains the width of the symbol *w* and the length of the symbol *l*. We can obtain the area map features *m*_1_ = *S*(*g*_1_, *q*) and *m*_2_ = *S*(*g*_2_, *q*). The formula *m*_1_⊕_M_*m*_2_ = *m*_3_ holds true; therefore, *m*_1_ and *m*_2_ can be properly matched when the following criterias are met: (1) |*x*_1_ − *x*_2_| = *k* × *w* (*k* is an integer), (2) |*y*_1_ − *y*_2_| = *n* × *l* (*n* is an integer), and (3) *g*_1_ and *g*_2_ satisfy Definition 1. In this formula, *m*_3_ can be expressed as *m*_3_ = *S*(*g*_1_⊕_*G*_*g*_2_, *q*).

**Fig 11:**
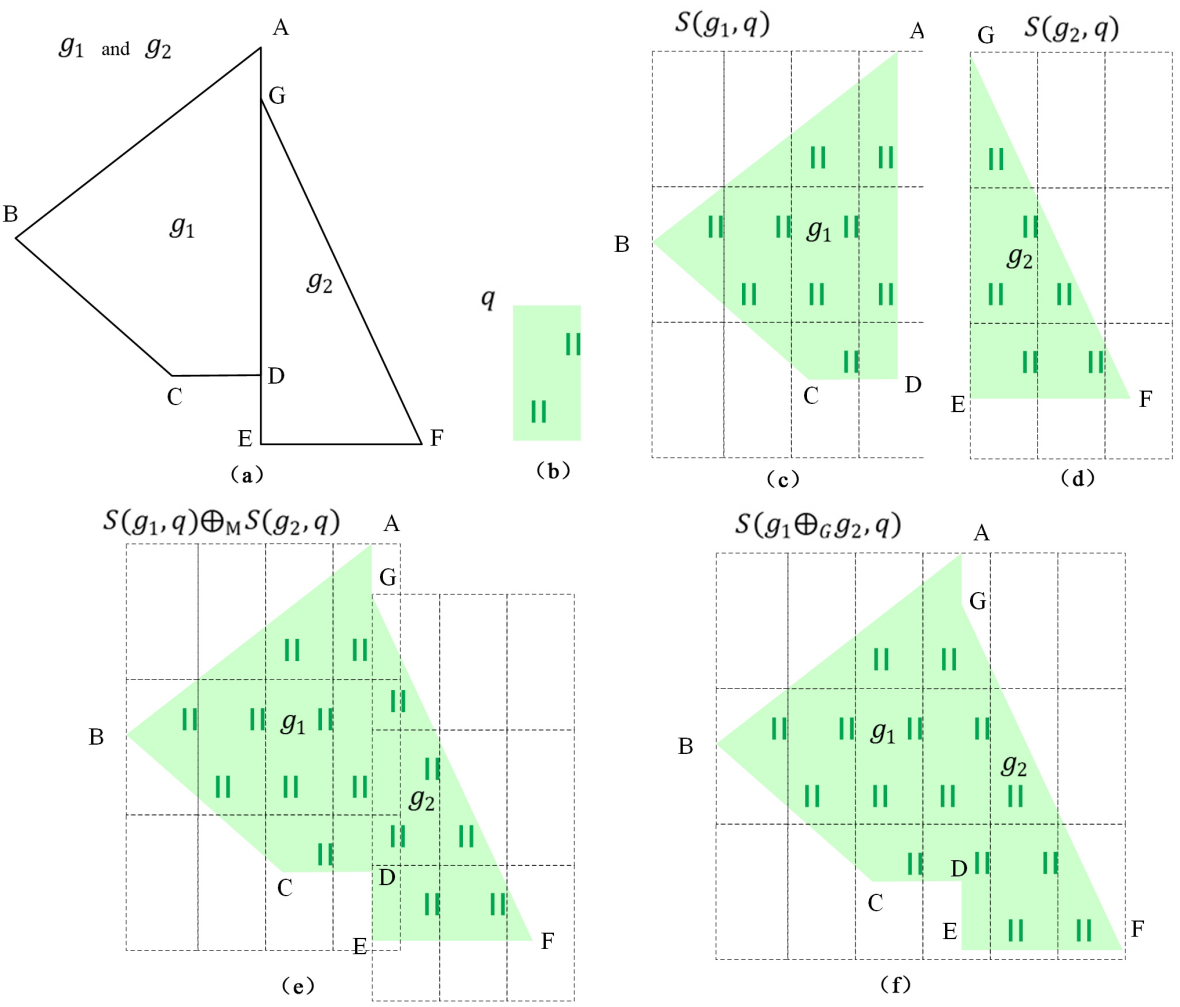
Example that does not satisfy Definition 6: (a) area geographical features *g*_1_ and *g*_2_, (b) grassland symbol *q*, (c) map features of *g*_1_, (d) map features of *g*_2_, (e) map feature *m*_1_⊕_M_*m*_2_, and (f) map feature *S*(*g*_1_⊕_*G*_*g*_2_, *q*).

**Fig 12:**
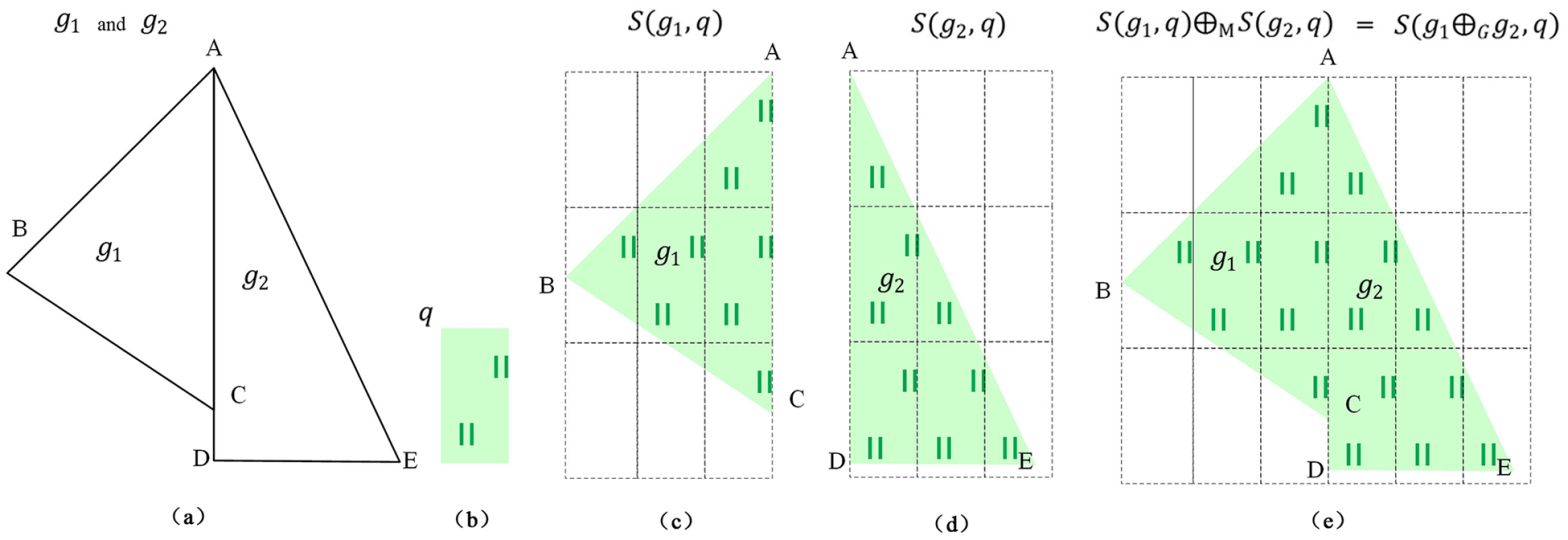
Example that satisfies Definition 6: (a) area geographical features *g*_1_ and *g*_2_, (b) grassland symbol *q*, (c) map features of *g*_1_, (d) map features of *g*_2_, (e) map features *m*_1_⊕_M_*m*_2_ and *m*_1_⊕_M_*m*_2_ = *S*(*g*_1_⊕_*G*_*g*_2_, *q*).

Some examples are shown in Figs 11 and 12 to explain Definition 6. As shown in Fig 11, the area features are *g*_1_ (polygon A-B-C-D) and *g*_2_ (polygon G-E-F). The symbol is *q*, and the symbol parameter is *λ*(*w***,** *l*). The map features of *g*_1_ and *g*_2_ are *m*_1_ = *S*(*g*_1_, *q*) and *m*_2_ = *S*(*g*_2_, *q*), respectively. The circumscribing rectangle of *g*_1_ and *g*_2_ can be divided by the corresponding symbol as the dashed boxes shown in Fig 11. Obviously, the areas *g*_1_ and *g*_2_ do not satisfy (1) |*x*_1_ − *x*_2_| = *k* × *w* (*k* is an integer) and (2) |*y*_1_ − *y*_2_| = *n* × *l* (*n* is an integer). Therefore, as shown in Fig 11, *S*(*g*_1_, *q*)⊕_M_ *S*(*g*_2_, *q*) is not equal to *S*(*g*_1_⊕_*G*_*g*_2_, *q*). In Fig 12, the areas *g*_1_ and *g*_2_ satisfy Definition 6 and *S*(*g*_1_, *q*)⊕_M_ *S*(*g*_2_, *q*) is equal to *S*(*g*_1_⊕_*G*_*g*_2_, *q*), as shown in Fig 12.

Based on Definition 5 and Definition 6, the tiled vector data model for area features is as follows:

$$g=\{\begin{array}{c} \Delta g,\text{ the fill pattern is color filling}\\ \Delta g{⊕}_{G}\sigma \text{, the fill pattern is color filling} \end{array}$$ (17)

In Formula (17), Δ*g* is the result of the area feature that is clipped by tiles and *σ* is an expansion feature of Δ*g* that allows *g* to satisfy the data model. The details of the expansion feature *σ* are illustrated in Section “Data organization method for area features”.

### Data organization based on the tiled vector data model

We now propose data organization methods for vector features based on the “Tiled vector data model for point, linear and area features” section. The results can avoid graphic conflicts and losses when they are symbolized. Moreover, the results can ensure the correctness and integrity of the geographical features. These features can be applied to user interactions and spatial analyses.

#### Data organization method for point features

Based on the data model for point features, the data organization method for point features consists of the following steps:

1. Obtain the circumscribing polygon of the map feature. If the circumscribing polygon does not intersect the target tile, skip the point.
2. If the circumscribing polygon of the map feature intersects the target tile, add the point to the feature set of the vector tile.

**Fig 13:**
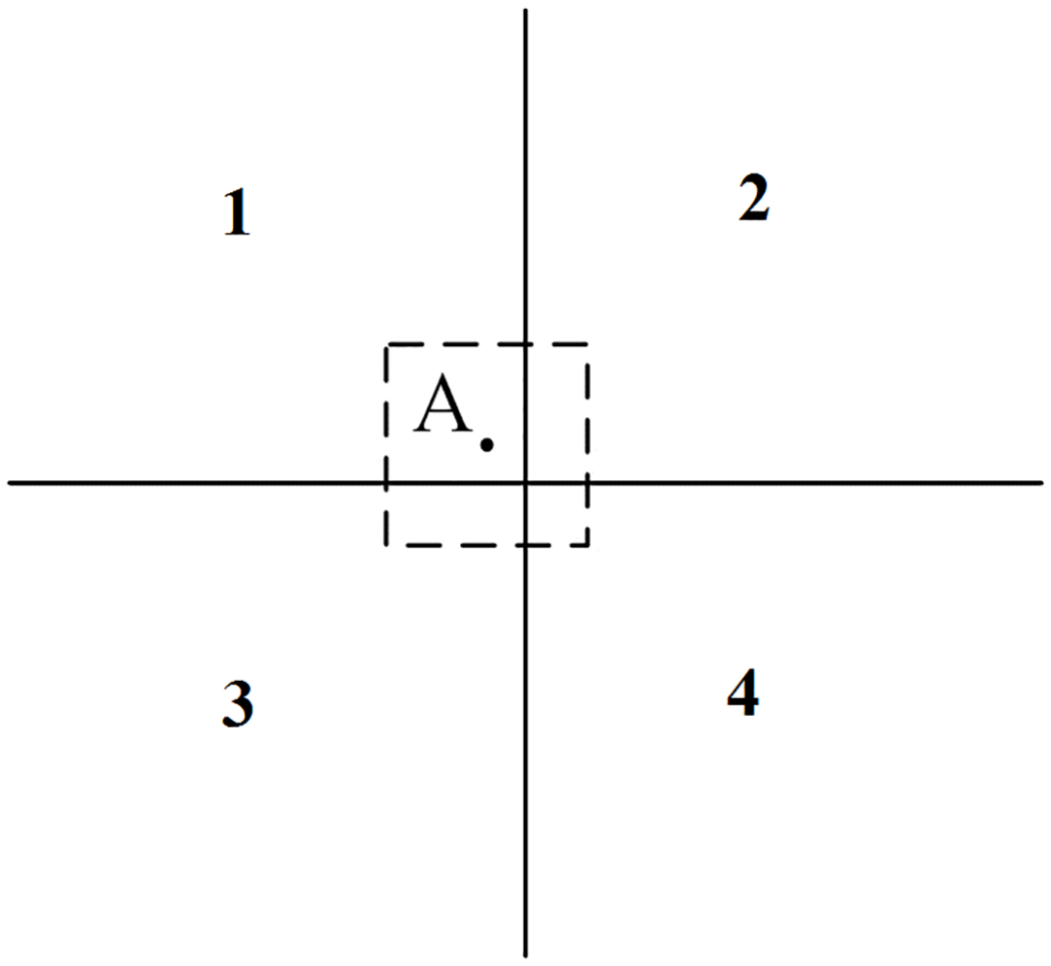
Example of organized point features.

An example of point feature data organization is shown in Fig 13. In the traditional data model for point features, only tile 1 contains Point A, which may cause graphic losses, as illustrated in Fig 2. Based on the proposed tiled vector data model for point features, tile 1 satisfies case 1, and tiles 2–4 satisfy case 2. Therefore, we added Point A to the feature set of all of the tiles (tiles 1–4).

#### Data organization method for linear features

**Fig 14:**
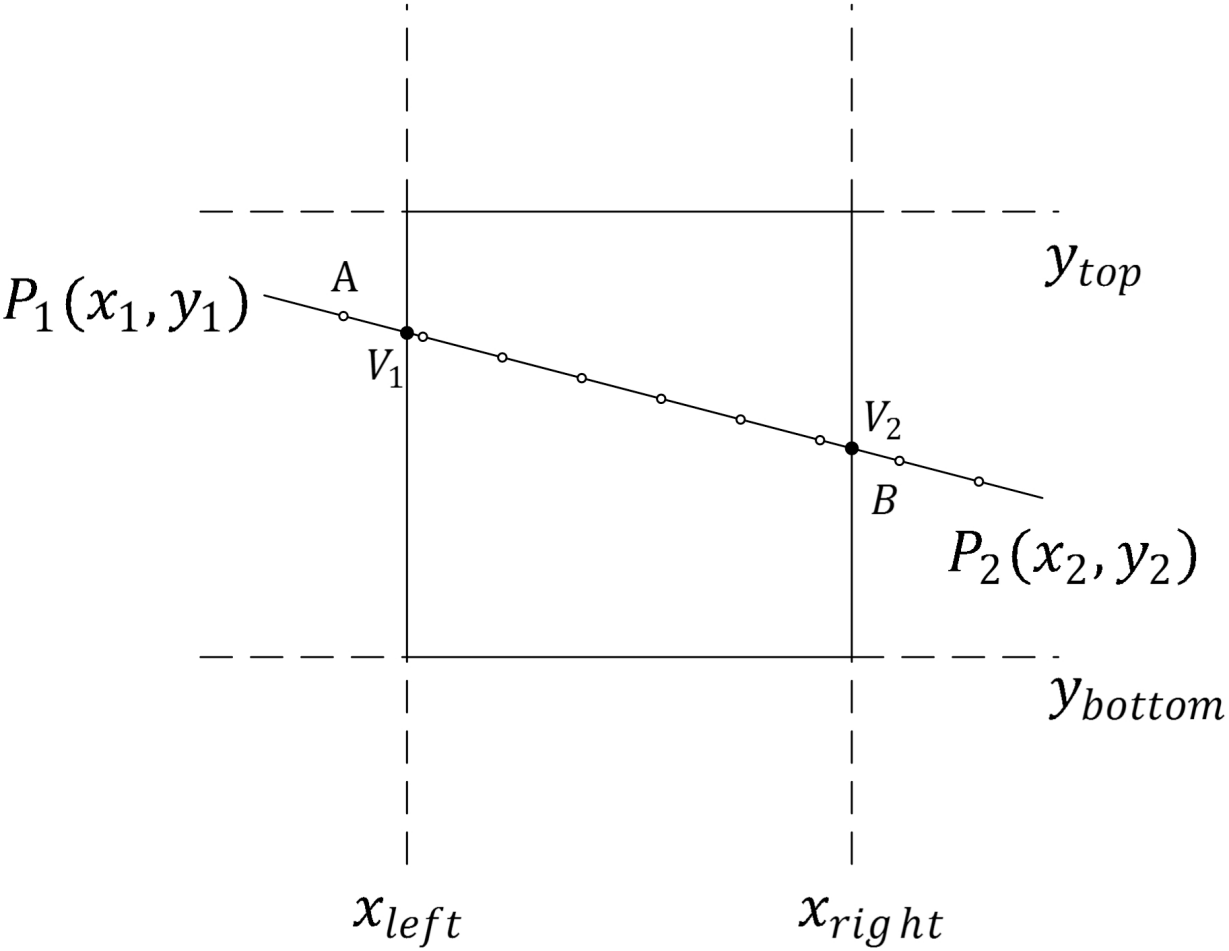
Example of organized linear features.

The preparation for processing linear features mainly includes the following steps. Step one is to obtain the corresponding symbol for the linear feature. Then, the linear feature is divided into many segments based on the length symbol. We fully exploit the rectangular characteristic of the tile. The Liang-Barsky [53] algorithm is appropriate for organizing lines. The Liang-Barsky algorithm is a parametric line clipping algorithm that clips straight lines against a standard rectangle. The algorithm operation process is simple because it only considers parameters. Coordinate operations are only performed when necessary. The Liang-Barsky algorithm can meet the requirements of the organization method and improves the computational efficiency. As shown in Fig 14, a linear feature ***P***_**1**_ **− *P***_**2**_ crosses the tile, and the steps for organizing linear features are as follows:

1. Divide a line into parts according to the length of the symbol. Uniform nodes are the hollow nodes in Fig 14.
2. Obtain the intersection points of the linear feature *P*_1_ − *P*_2_ and tile, called *V*_1_ and *V*_2_, respectively. In the traditional model, the saved feature in the tile is *V*_1_ − *V*_2_. However, this feature may cause graphic conflicts and losses along the borders of tiles.
3. Based on the tiled vector data model for linear features (Formula (11)), the model of saved features is $g=\sigma_{1}{⊕}_{G}\Delta g_{1}{⊕}_{G}\wedge_{i=1}^{n}g_{i}^{\lambda }{⊕}_{G}\Delta g_{2}{⊕}_{G}\sigma_{2}$. $\Delta g_{1}{⊕}_{G}\wedge_{i=1}^{n}g_{i}^{\lambda }{⊕}_{G}\Delta g_{2}$ is the linear feature *V*_1_ − *V*_2_ in this case, and we must add the expansions *σ*_1_ and *σ*_2_ to *V*_1_ − *V*_2_. Finally, A − *V*_1_ and *V*_2_ − *B* are *σ*_1_ and *σ*_2_, and the saved feature in the tile is A − *V*_1_ − *V*_2_ − *B*.

**Fig 15:**
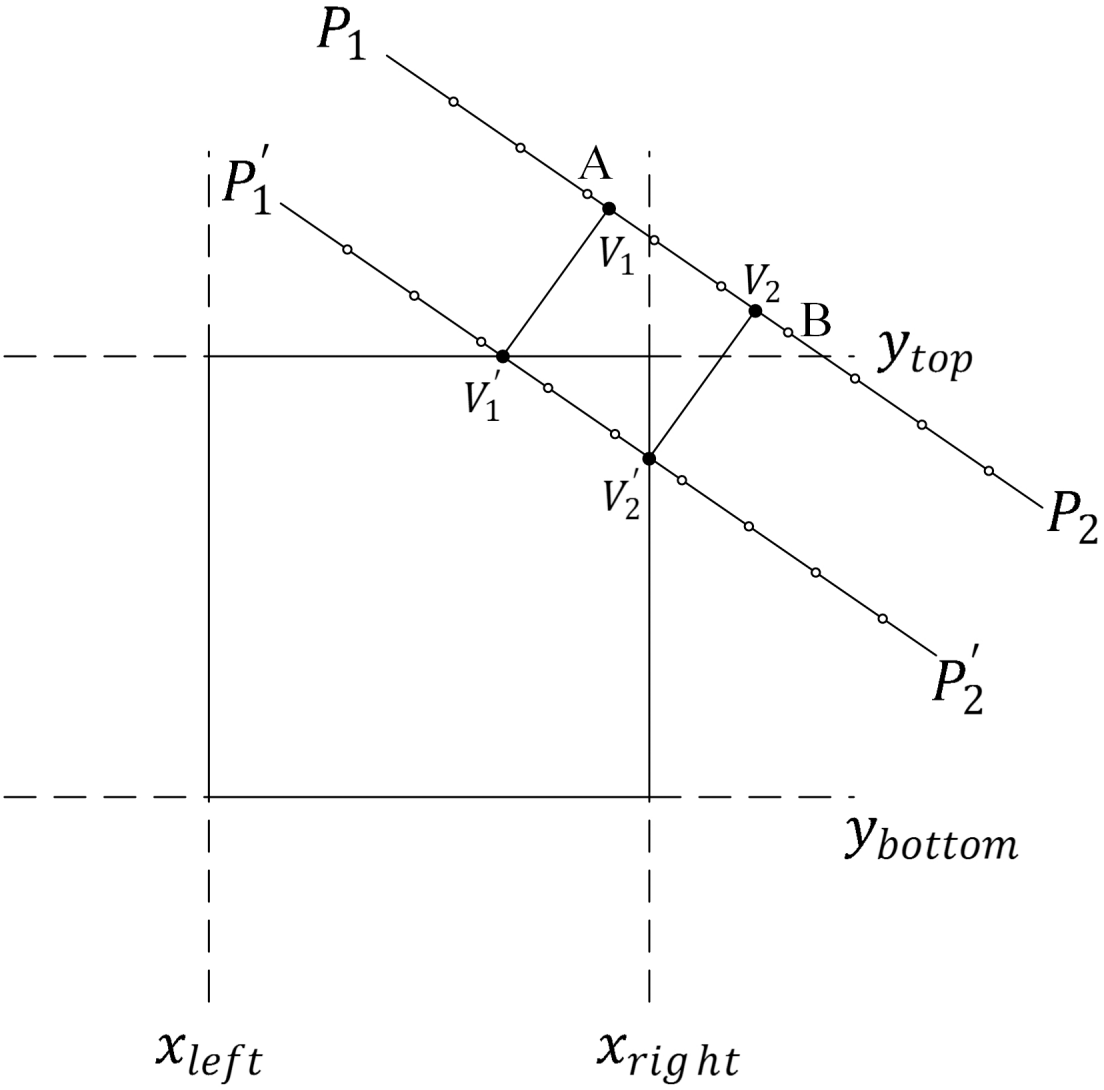
Special case of organizing linear features.

Another case that should be considered is a linear feature that does not intersect the tile alongside a map feature that does intersect the tile. This case may result in the loss of the map feature. Concrete details are shown in Fig 15. A method to manage such situations is as follows:

1. Divide the linear feature *P*_1_ − *P*_2_ into sections according to the length of the symbol. Uniform nodes are the hollow nodes in Fig 15.
2. According to the width of the feature symbol, move the feature to the direction of the tile and obtain new features, such as $P_{1}^{\prime }-P_{2}^{\prime }$ in Fig 15.
3. Find the intersection points of $P_{1}^{\prime }-P_{2}^{\prime }$ and the tile, called $V_{1}^{\prime }$ and $V_{2}^{\prime }$, respectively, and then measure the perpendiculars of $V_{1}^{\prime }$ and $V_{2}^{\prime }$ with regard to *P*_1_*P*_2_. The corresponding foot points are called *V*_1_ and *V*_2_, which can be regarded as the intersections of the features and tiles.
4. Combined with the equal nodes of the symbols, expand the feature based on the tiled vector data model for linear features (Formula (11)). Finally, the saved feature in the tile is A − *V*_1_ − *V*_2_ − *B*.

#### Data organization method for area features

**Fig 16:**
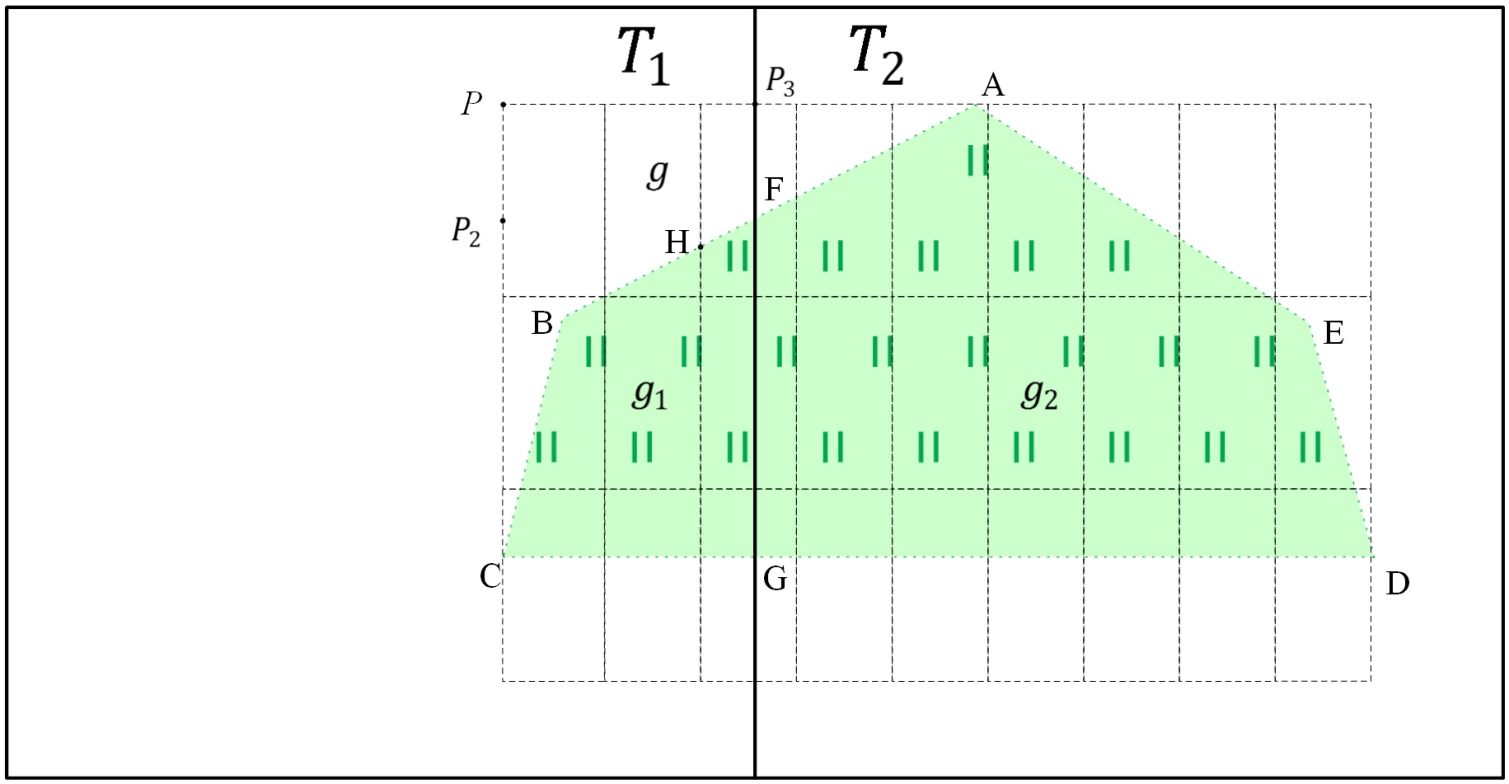
Example of organized area features.

In this section, the grassland symbol was chosen as an example to illustrate data organization for area features. As shown in Fig 16, two tiles are marked *T*_1_ and *T*_2_. An area feature *g* (polygon A-B-C-D-E) intersects the tiles and is clipped by the tiles into *g*_1_ (polygon F-B-C-G) and *g*_2_ (polygon A-F-G-D-E). The symbol for grassland is *q*, and the symbol parameter is *λ*(*w*, *l*). The map features of *g*, *g*_1_ and *g*_2_ are *m = S*(*g*, *q*), *m*_1_* = S*(*g*_1_, *q*), *m*_2_* = S*(*g*_2_, *q*) respectively. The symbolization starting points of *g*, *g*_1_ and *g*_2_ are located in *P*(*x*, *y*), *P*_2_(*x*_2_, *y*_2_) and *P*_3_(*x*_3_, *y*_3_), respectively. The circumscribing rectangle can be divided by the corresponding symbol into the dashed boxes in Fig 16. The areas *g*_1_ and *g*_2_ do not satisfy Definition 6; therefore, *m*_1_⊕_M_*m*_2_ is not equal to *m* and the map features cannot be matched along the borders of the tiles.

Based on the tiled vector data model for area features in Section “Tiled vector data model for area features”, the key of this data organization is constructing the corresponding expansion features *σ*_1_ and *σ*_2_ for *g*_1_ and *g*_2_. However, for the feature *g*_1_, *x* = *x*_1_, *y* − *y*_1_ ≠ *n* × *l* (*n* is an integer). Therefore, we must find a point $P_{1}^{\prime }\left(x_{1}^{\prime },y_{1}^{\prime }\right)$ from *g*_2_ so that $P_{1}^{\prime }$ satisfies the following formula:

$$\left|\text{ y}-y_{1}^{\prime }\right|=n\times l\;(n\text{ is an integer})$$ (18)

The expansion feature for *g*_1_ is constructed by $P_{1}^{\prime }$. In this case, the vertex A can satisfy the formula and minimize the area of the expansion feature. Therefore, the expansion feature *σ*_1_ is the polygon A-F-G. Finally, we add the polygon A-F-B-C-G to the feature set of tile *T*_1_. Similarly, we must find a point $P_{2}^{\prime }\left(x_{2}^{\prime },y_{2}^{\prime }\right)$ for the feature *g*_2_ from *g*_1_ so that $P_{2}^{\prime }$ satisfies the following formula:

$$\left|\text{ x}-x_{2}^{\prime }\right|=n\times w\;(n\text{ is an integer})$$ (19)

The vertex H can satisfy the formula, and the expansion feature *σ*_2_ is the polygon F-H-G. Finally, we add the polygon A-F-H-G-D-E to the feature set of tile *T*_2_.

## Experiments

The proposed tiled vector data model are illustrated experimentally by using the data organization methods in Section “Data organization based on the tiled vector data model”.

### Experiment setting and implementation

The experimental platform of this article is a personal computer (PC) with an Intel i5-4670T central processing unit (CPU) with 2.3GHz, 4 processor counts, and 8.00 GB random access memory (RAM) running the Microsoft Windows 7 Ultimate x64 operating system. All the experiments were implemented in Desktop as our team is experienced in representing symbols with this software. The generating tile program used to implement the proposed tiled vector data model was realized using Visual C#.NET and ArcEngine of ArcGIS. ArcEngine was used for accessing spatial data as well as a tool for geometric calculation. The map explorer program was implemented using Visual C#.NET and a graphics rendering library (graphics device interface plus (GDI+)).

The experimental data were obtained from a 1:10,000 scale electronic map, including various types of geographical features, such as “main street”,”subway”,”railway”,”building”,”canal”,”vegetation”,”school” and”hospital”. As the objective of the experiment was to test the proposed tiled vector data model, all the features are not involve in map generalization. All of the features were saved as shapefile (*.shp). Then, we used the data model proposed in Section “Tiled vector data model for point, linear and area features” and organized the data using the methods outlined in Section “Data organization based on the tiled vector data model”. The organization results for each tile can be saved in any format for decoding (we saved the vector tile in the form of shapefile in the article). Naming rules were based on the rows and columns of corresponding tiles. Thus, the name of the tile in row 5, column 4 was “5_4”. The advantage of this convention is its convenience for querying and its ability to effectively and accurately finding the target tiles.

**Fig 17:**
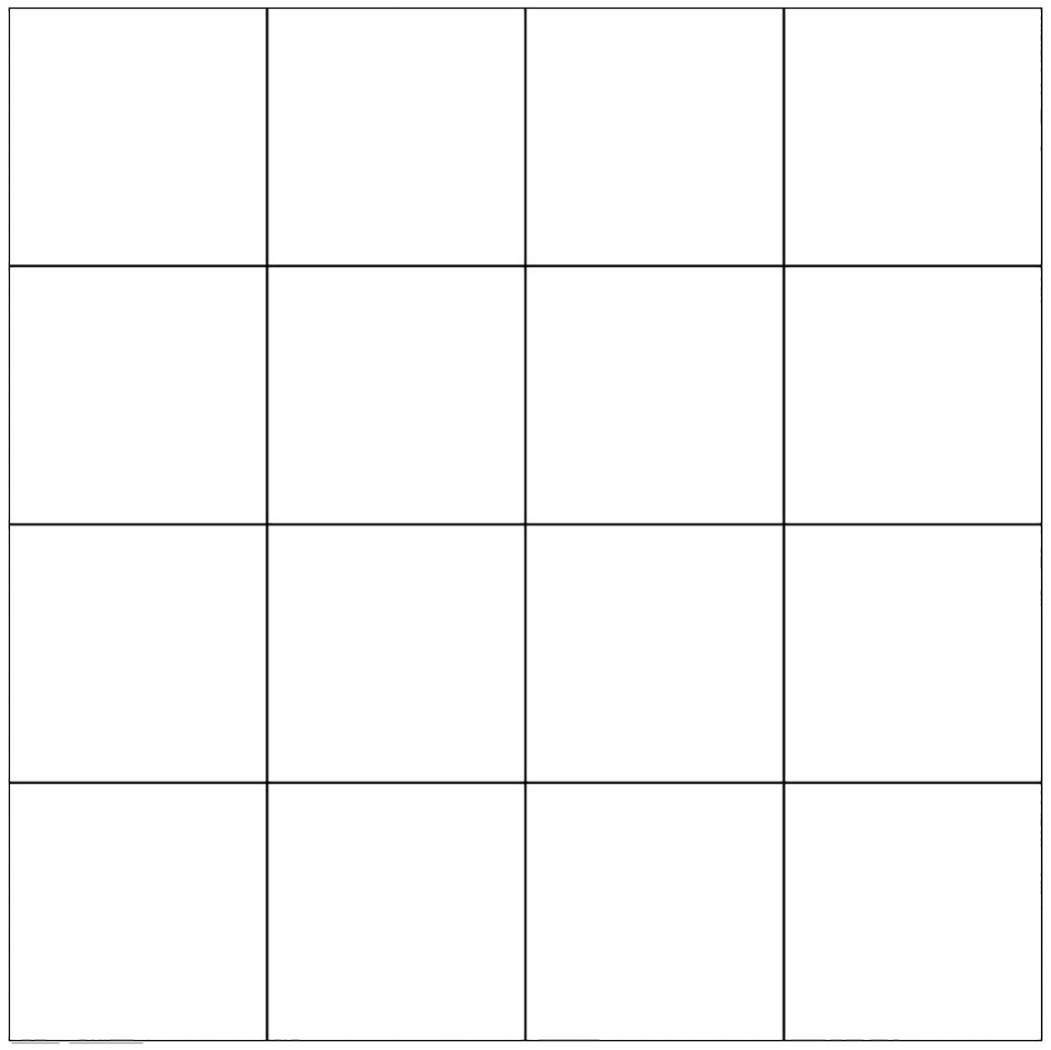
Distribution form of map explorer tiles.

The map explorer consisted of 16 tiles. The distribution form of the tiles on the map explorer is shown in Fig 17. All the functions of the map explorer, such as initializing the symbol library, symbolizing the features and viewing the map, were implemented in this experiment. Importantly, the map explorer had to initialize the symbol library before the tiles were loaded [54]. Then, tiles could be loaded into the map explorer and the map browser could render the map features in each tile as E-maps.

### Experimental results

#### Map visualization

**Fig 18:**
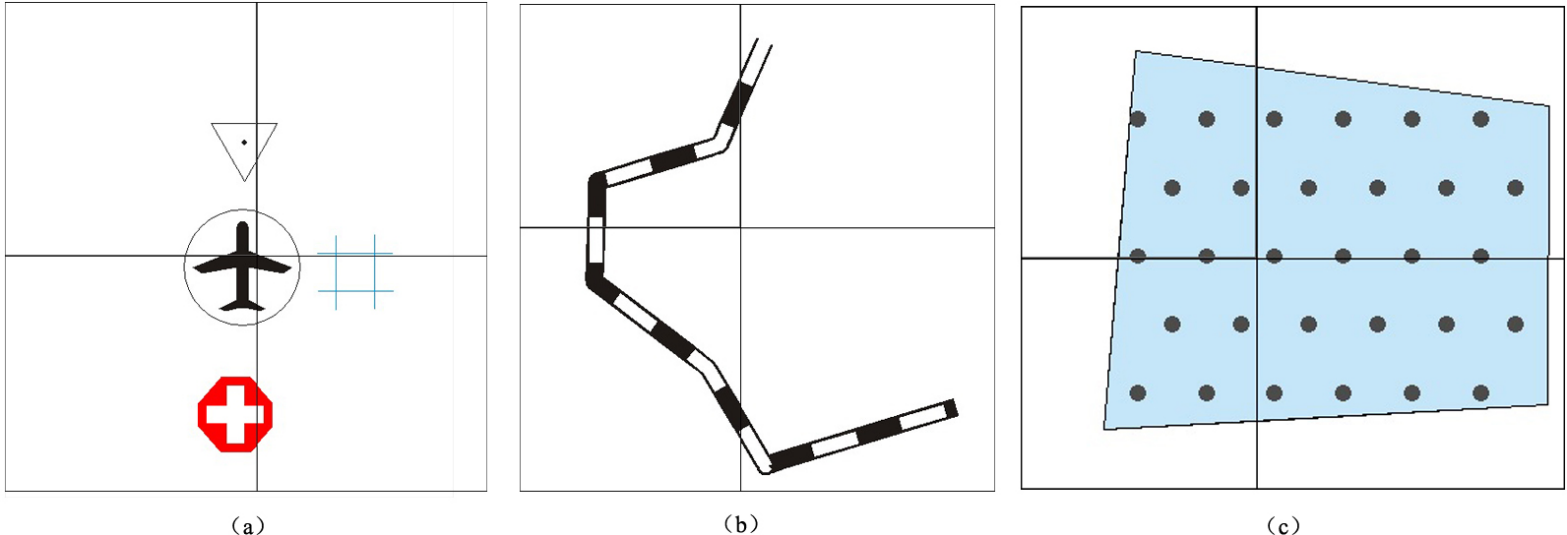
Rendering results based the proposed data model for feature (a) points, (b) lines and (c) areas.

Examples of the three basic types of features were used to verify the feasibility of the proposed tiled vector data model presented in Section “Tiled vector data model for point, linear, area features”. As shown in Fig 18, the results for each type of feature showed good performances, demonstrating the proposed data model was able to effectively solve the problems of map feature matching at the borders of tiles that are shown in Fig 2.

**Fig 19:**
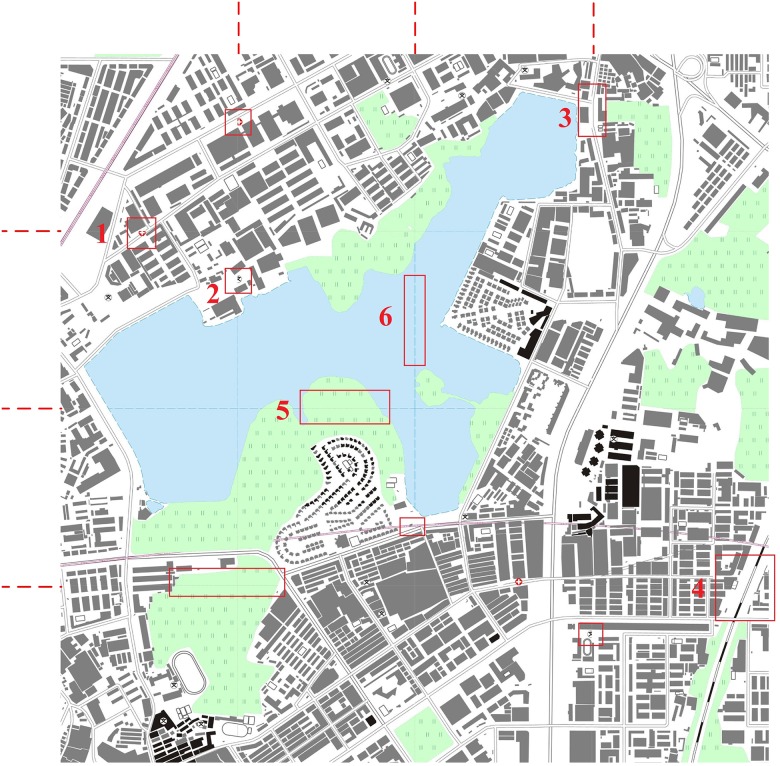
E-map based on vector tiles at the 1:10,000 scale based on the traditional data model.

**Fig 20:**
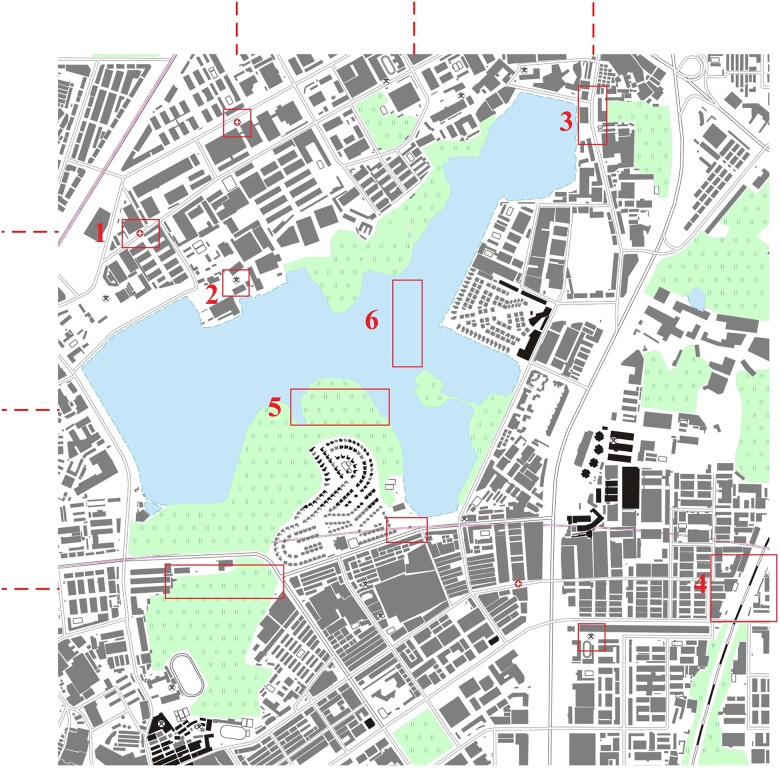
E-map based on vector tiles at the 1:10,000 scale based on the proposed data model.

**Fig 21:**
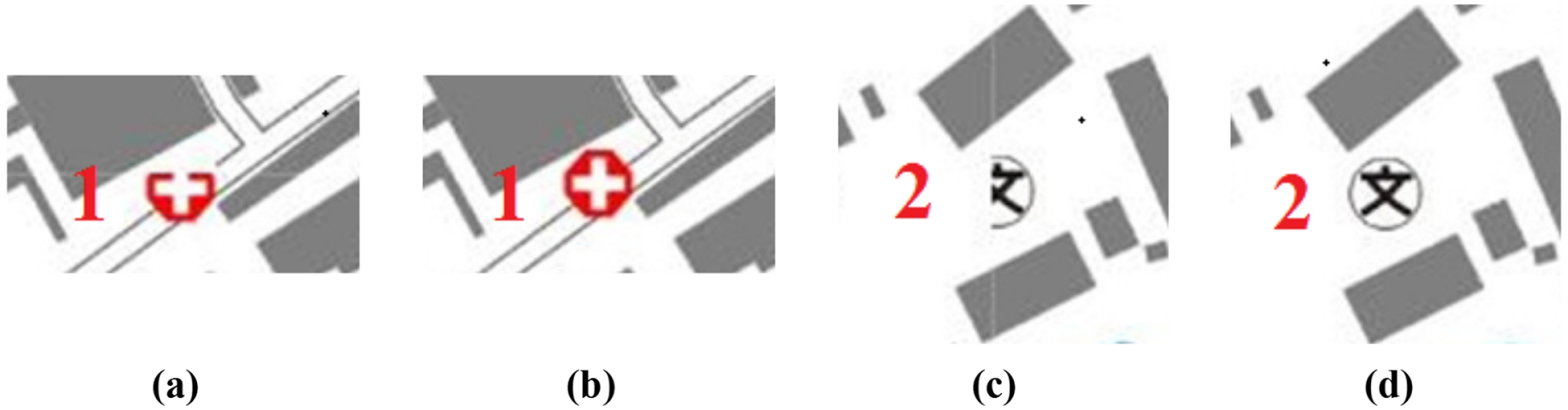
Detailed comparisons of the point features: (a) and (c) are from Fig 19, and (b) and (d) are from Fig 20.

**Fig 22:**
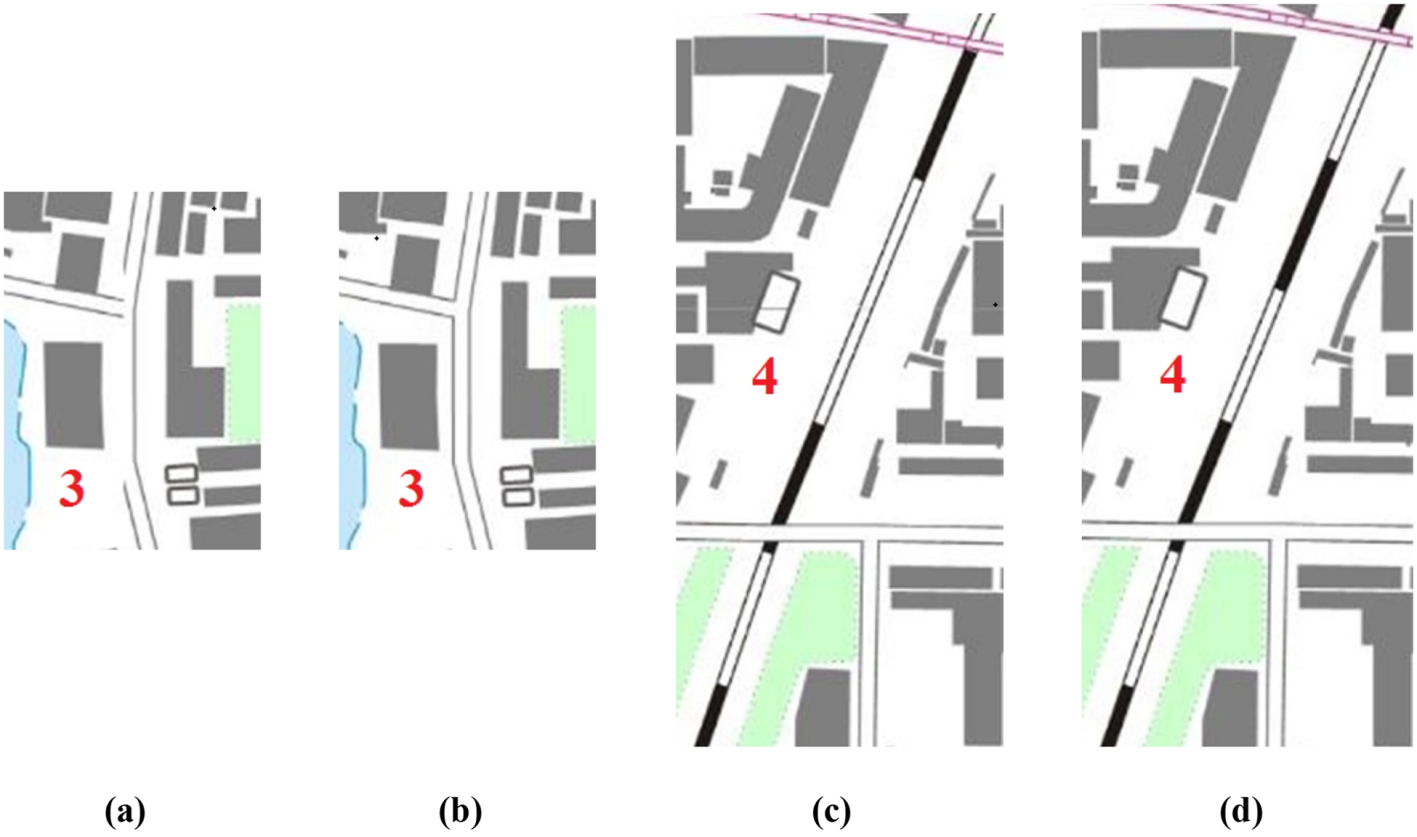
Detailed comparisons of the linear features: (a) and (c) are from Fig 19, and (b) and (d) are from Fig 20.

**Fig 23:**
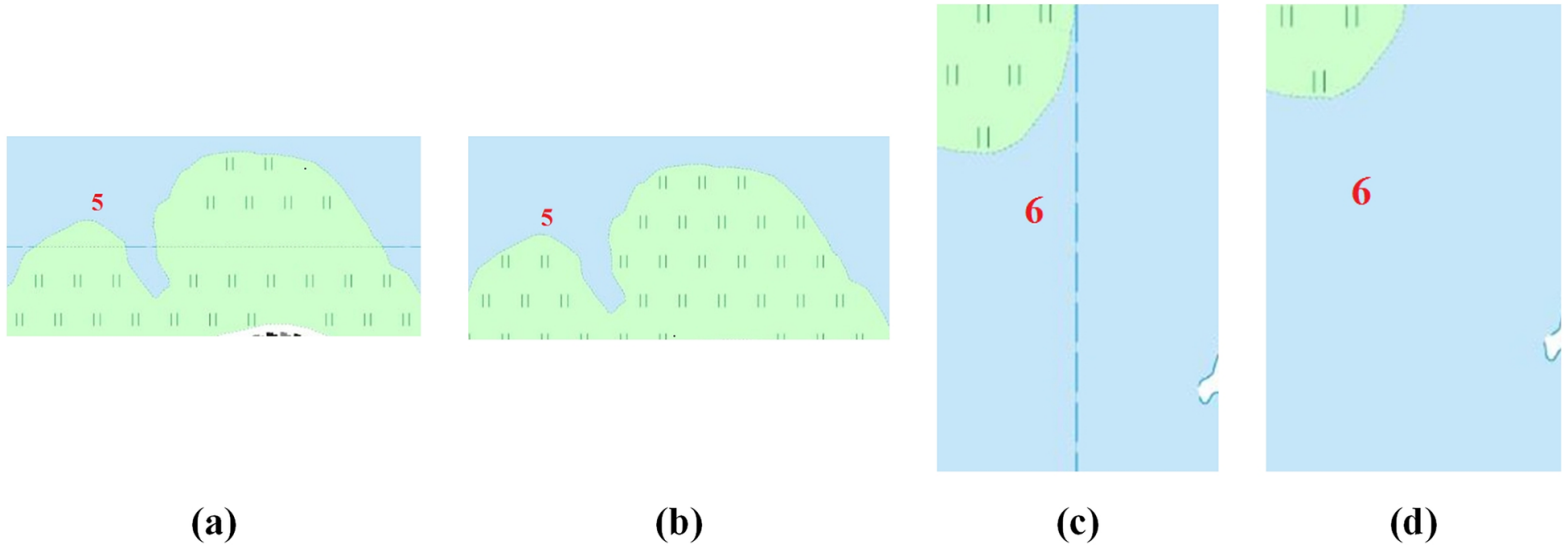
Detailed comparisons of the area features: (a) and (c) are from Fig 19, and (b) and (d) are from Fig 20.

**Fig 24:**
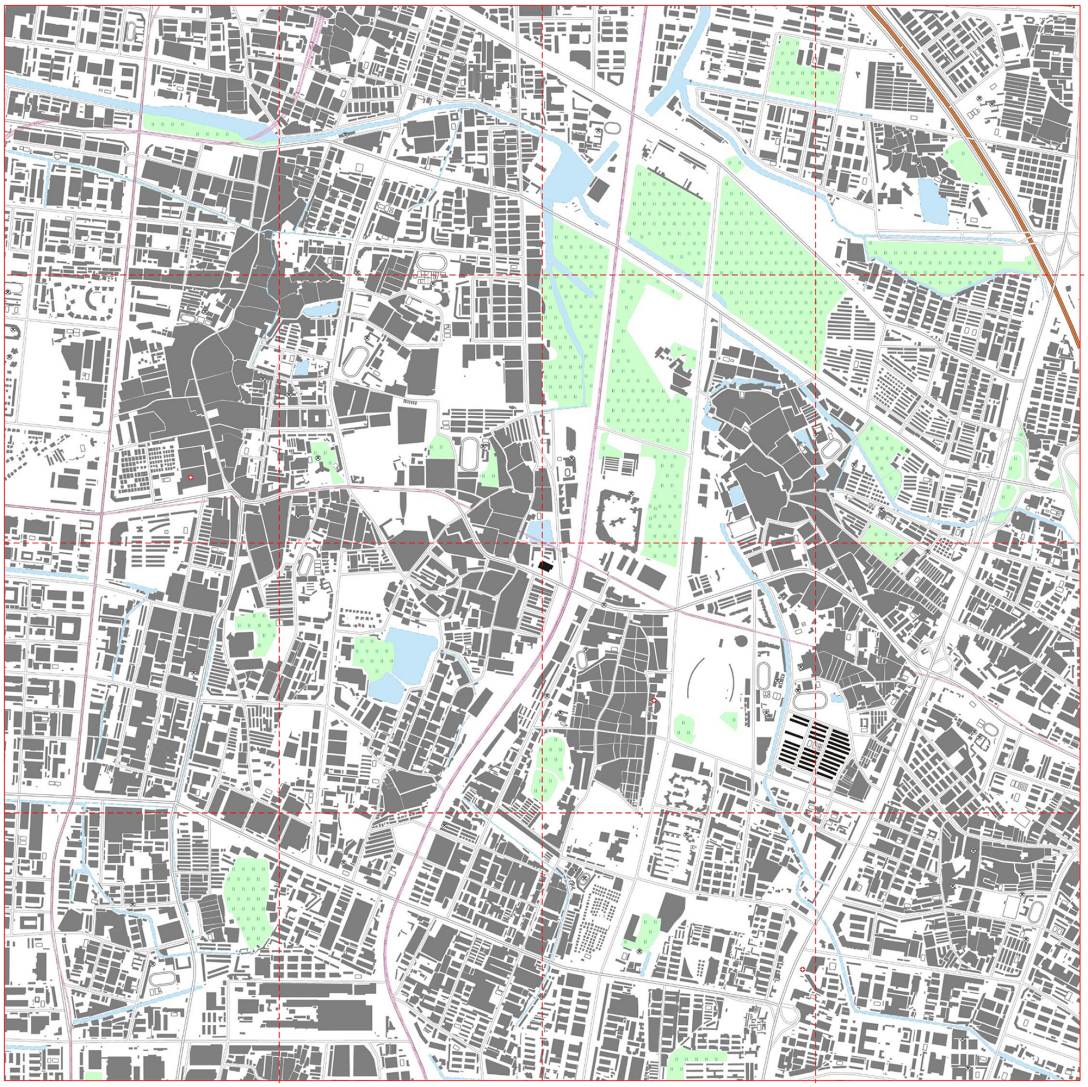
E-map based on vector tiles at the 1:20,000 scale based on the proposed data model.

To further evaluate the strength of the proposed model, we generated and visualized vector tiles using different data models in different scales. Figs 19 and 20 show the experimental results that using the traditional data model and the proposed data model, respectively, based on the same experimental data at the 1:10,000 scale. The red dotted lines in Figs 19 and 20 are the frames of each tile. The graphic conflicts and losses that are caused by the traditional data model are marked in Fig 19, whereas a perfect effect is displayed in Fig 20, which does not show the problems that are associated with graphic conflicts and losses. Detailed comparisons of typical examples with serial numbers are shown in Figs 21–23. Fig 24 shows the results at the 1:20,000 scale based on the proposed data model. Figs 20 and 24 show that the map features at the borders of the vector tiles are well matched. The good visualization results demonstrate that the tiled vector data model for geographical features is feasible for different map data sets and at different scales.

#### Generating efficiency comparison

The running times of generating tiles for raster tiles and vector tiles based on the traditional model and the proposed model are shown in Table 1. The experiments assess different sizes of data and all the experiments were performed 10 times to obtain the average time cost to reduce the randomness of the experiments. From Table 1, we can see that generating vector tiles costs more than generating raster tiles. Because the method of generating raster tiles is only image clip while generating vector tiles needs lots of geometric computations. However, compared with raster tiles, vector tiles allow users to interact with the system and perform spatial analysis. The efficiency of generating vector tiles based on the traditional model was better than that based on the proposed model. The result is due to the structure of the proposed model, which is more complicated than the traditional model and requires additional geometric computations. Nevertheless, the main focus of this study is to eliminate graphic conflicts and losses, and such elimination is the unique advantage of the proposed data model compared with the traditional data model. Furthermore, the vector tiles can be pre-generated at the server-side. Therefore, this loss in efficiency of generating tiles is acceptable.

**Table 1:** Time cost analysis of generating tiles for the three models (in seconds).

Features (Tiles)	Raster tiles			Vector tiles based on the traditional model			Vector tiles based on the proposed model		
	Time cost	Visual continuity	Support interactivity	Time cost	Visual continuity	Support interactivity	Time cost	Visual continuity	Support interactivity
97074(1320)	65.8	Yes	No	3740.78	No	Yes	5601.17	Yes	Yes
5962(42)	0.7	Yes	No	53.478	No	Yes	74.87	Yes	Yes
2098(16)	0.2	Yes	No	22.13	No	Yes	30.15	Yes	Yes

#### Rendering efficiency comparison

Table 2 tile types (raster tiles, vector tiles based on the traditional model and vector tiles based on the proposed model). The time of rendering raster tiles can be ignored because raster tiles are images. Due to the complex structure of proposed model, rendering vector tiles based on this model required slightly more time than that required by the traditional model, but this increase in time cost had no effect on the performance of the browsing map.

**Table 2:** Time cost analysis of rendering tiles for the three models (in seconds).

Features (Tiles)	Raster tiles			Vector tiles based on the traditional model			Vector tiles based on the proposed model		
	Time cost	Visual continuity	Support interactivity	Time cost	Visual continuity	Support interactivity	Time cost	Visual continuity	Support interactivity
5962(42)	-	Yes	No	1.49	No	Yes	1.78	Yes	Yes
2098(16)	-	Yes	No	0.51	No	Yes	0.58	Yes	Yes

As this article focuses on proposing a tiled vector data model for geographical features, optimized methods of generating tiles and rendering tiles are beyond the scope of this paper. The running times in Tables 1 and 2 are only reference values.

## Discussion and conclusions

E-maps are widely applied in many fields. Most current applications of E-maps are implemented based on raster tiles or vector tiles that are generated based on the traditional data model. This article summarizes previous literatures and explains why the traditional data model is inappropriate for vector tiles. It then proposes a novel tiled vector data model for geographical features. The proposed data model is superior to traditional methods in eliminating visual discontinuities and conflicts at the tile borders. In additional, we verify the proposed model is theoretically correct and feasible by formula derivation and several experiments. The conclusions can be summarized as follows:

1. This paper proposed a theoretical framework for vector tile technology. We define some operators and definitions for geographical features and map features. Model derivations based on mathematics are used to illustrate the shortcomings of the traditional data model and testify the feasibility and validity of the proposed tiled data model. The proposed tiled data model is an advancement in vector tile technology of E-maps.
2. The proposed model is tested by performing several experiments using ground-truth data in different scales on a desktop platform. The experimental results indicate that the proposed vector data model can solve visual conflicts and discontinuities for all types of geographical features that is the greatest weakness of the traditional data model. The proposed model can be used as reference to solve similar problems for E-maps of mobile and web clients.
3. Although the efficiency of generating and rendering tiles is lower for the proposed model than for the traditional model, the efficiency is acceptable considering the proposed model's complicated structure. Moreover, the cost of generating tiles can be offset by pre-generating all of the vector tiles before rendering the tiles. For rendering tiles, the time costs for the proposed model and the traditional model are almost equal, although the proposed model has an unique advantage. The efficiency comparisons reveals that the proposed model solve the conflicts problem while sacrificing very little time cost, which is significant for applications of vector tile technology.

This work has a good potential for contributing to enlarge the application scope of E-maps but still suffers from several limitations, which could be addressed in future research. First, the proposed model is only valid for maps with static symbols. The design of map symbols is open, and certain symbols that are cognitively effective for communication but might be realized by special procedures, such as dynamic symbols, which are especially useful for E-maps. Most dynamic symbols are dynamic or Flash images, and their symbol parameters (the length or the width of symbols) cannot be obtained directly. In future work, we will create an extension for the library to support dynamic symbols so that we can obtain the symbol parameter easily. Second, the proposed data model is more complicated than the traditional model, and the data size of vector tiles based on the proposed model is slightly larger. A balance between data transmission and cartographical rendering is critical for implementing the model. We will use “Protocolbuffer Binary Format” (PBF) as the encoding format for vector tiles rather than GeoJSON in future work. PBF is a high-compact format, and files encoded in PBF are much smaller than those encoded in GeoJSON. Using this technology will improve the transmission performance. Finally, real-time interactivity and spatial analysis have not been considered in this article. The geographical features are clipped into several vector fragments and stored in different vector tiles. These vector fragments cannot be used for interaction and spatial analysis, which are two merits of vector data and depend on reconstructing vector fragments and storing entire geographical features. The key to reconstruction is to seek out fragments and obtain the vertex series according to the chained relationships among vector tiles. These aforementioned limitations are the focus of our future work.

## Supporting information

S1 File Symbol library. (XML)

S2 File Experimental dataset. (ZIP)
